# A Generalized Interpolation Material Point Method for Shallow Ice Shelves. 2: Anisotropic Nonlocal Damage Mechanics and Rift Propagation

**DOI:** 10.1029/2020MS002292

**Published:** 2021-08-24

**Authors:** Alex Huth, Ravindra Duddu, Ben Smith

**Affiliations:** ^1^ Department of Earth and Space Sciences University of Washington Seattle WA USA; ^2^ Now at Atmospheric and Oceanic Sciences Princeton University Princeton NJ USA; ^3^ Department of Civil and Environmental Engineering Vanderbilt University Nashville TN USA; ^4^ Department of Earth and Environmental Sciences Vanderbilt University Nashville TN USA; ^5^ Applied Physics Laboratory Polar Science Center University of Washington Seattle WA USA

**Keywords:** Damage, fracture, glaciology, ice shelves, material point method, particle method

## Abstract

Ice shelf fracture is responsible for roughly half of Antarctic ice mass loss in the form of calving and can weaken buttressing of upstream ice flow. Large uncertainties associated with the ice sheet response to climate variations are due to a poor understanding of these fracture processes and how to model them. Here, we address these problems by implementing an anisotropic, nonlocal integral formulation of creep damage within a large‐scale shallow‐shelf ice flow model. This model can be used to study the full evolution of fracture from initiation of crevassing to rifting that eventually causes tabular calving. While previous ice shelf fracture models have largely relied on simple expressions to estimate crevasse depths, our model parameterizes fracture as a progressive damage evolution process in three‐dimensions (3‐D). We also implement an efficient numerical framework based on the material point method, which avoids advection errors. Using an idealized marine ice sheet, we test the creep damage model and a crevasse‐depth based damage model, including a modified version of the latter that accounts for damage evolution due to necking and mass balance. We demonstrate that the creep damage model is best suited for capturing weakening and rifting over shorter (monthly/yearly) timescales, and that anisotropic damage reproduces typically observed fracture patterns better than isotropic damage. Because necking and mass balance can significantly influence damage on longer (decadal) timescales, we discuss the potential for a combined approach between models to best represent mechanical weakening and tabular calving within long‐term simulations.

## Key Points

- Our shallow‐shelf creep damage model can represent the full evolution of ice shelf fracture from crevasse initiation to tabular calving
- Strongly anisotropic creep damage produces sharp, arcuate rift patterns more consistent with observations than isotropic creep damage
- The zero‐stress damage model poorly captures rifting, but a modified form accounts for damage evolution due to mass balance and necking

## 1 Introduction

Fracture of ice shelves strongly impacts the evolution of the Antarctic Ice Sheet and its interaction with climate. Approximately half of ice mass loss is attributed to fracture‐induced calving, while the other half is attributed to ocean‐driven basal melting (Depoorter et al., 2013; Paolo et al., 2015; Rignot et al., 2013). Furthermore, mechanical weakening associated with fracture processes can decrease ice shelf buttressing of upstream grounded ice flow into the ocean, leading to sea level rise (e.g., Borstad et al., 2013; MacGregor et al., 2012). For example, the Antarctic glaciers that will likely contribute the most to sea level rise in the next centuries, Pine Island and Thwaites, are buttressed by ice shelves that contain only a limited region of ice that can be lost or weakened without dynamic consequences that would lead to increased mass loss from the grounded ice sheet (Fürst et al., 2016). In extreme cases, fracture can eliminate buttressing entirely if full ice shelf collapse occurs, as it did when the Larsen B Ice Shelf collapsed over a period of just six weeks in 2002, likely due to hydrofracture (Scambos et al., 2004) related to surface meltwater ponding enabled by rising surface air temperatures. Fracture is also interconnected with climate through ocean processes. Ocean driven basal‐melting of ice shelves can cause thinning that makes ice shelves more vulnerable to fracture (Liu et al., 2015; Shepherd et al., 2003). In turn, calved tabular icebergs can alter ocean circulation (e.g., Cougnon et al., 2017; Robinson et al., 2010; Stern et al., 2015, 2016).

The importance of ice shelf fracture processes to ice sheet and climate dynamics motivates their incorporation into prognostic flow models of ice sheet‐ice shelf systems to better assess ice shelf stability and project ice sheet response to climate change. An efficient, accurate, and commonly used ice flow model for these systems is the shallow shelf approximation (SSA), a two‐dimensional (2‐D) vertically integrated form of the incompressible Stokes equations (see Section 2.1). Prognostic representation of fracture in SSA models has ranged from simple calving parameterizations to explicitly modeling fracture evolution and its feedback on flow using damage variables. For calving alone, reasonable ice front positions have been obtained by parameterizing smooth calving rates (e.g., Alley et al., 2008; Levermann et al., 2012) or attempting to track crevasse depths over time, where crevasses are assumed to propagate to the depth where the horizontal Cauchy (total) stress equals zero (e.g., Nick et al., 2010, 2013; Nye, 1957; Pollard et al., 2015). This “zero‐stress” approach assumes crevasse depths are in equilibrium with the stress field, and has been further developed into damage models that may be used with the SSA (Bassis & Ma, 2015; Sun et al., 2017). Other SSA damage models (Albrecht & Levermann, 2012, 2014; Borstad et al., 2016) do not explicitly track crevasse depths. Borstad et al. (2016) formulated an SSA damage model by fitting a relationship between stress and damage fields inferred from observations of Larsen B Ice Shelf, but it was mostly successful near the ice shelf margins only and did not capture rifting. Albrecht and Levermann (2012, 2014) used a different SSA damage model that tested a variety of *ad hoc* measures for initiating fracture, but this model also did not capture rifting, and was only sufficient for broadly capturing the feedback between flow dynamics and fracture‐induced weakening.

While the zero stress model is easy to incorporate into ice sheet system models, it is not thermodynamically based, unlike linear elastic fracture mechanics (LEFM) models. Crevasse/rift propagation in LEFM models is based on the stress intensity factor and fracture toughness of ice, which are inherently related to strain energy release rate (where rate indicates per unit crack length) and fracture energy (i.e., the surface energy associated with the crack face), respectively. In the literature, analytical LEFM models were applied to estimate the penetration depth of crevasses (Rist et al., 2002; Smith, 1976; van der Veen, 1998a, 1998b; Weertman, 1973) and to study rift propagation in ice shelves (Hulbe et al., 2010; Larour et al., 2004a, 2004b; Lipovsky, 2018, 2020; Plate et al., 2012). Moreover, analytical LEFM models were combined with ice flow models to predict iceberg calving (Krug et al., 2014; Yu et al., 2017) and to investigate ice shelf stability (Lai et al., 2020). However, analytical LEFM models rely on weight functions to evaluate the stress intensity factor that are only applicable for idealized geometries (e.g., rectangular plates) and boundary conditions (e.g., traction free or symmetric edge), and when material nonlinearities (e.g., nonlinear viscous rheology) can be neglected. Therefore, their relevance to iceberg calving of real glaciers and ice shelves can be somewhat limited (Jiménez & Duddu, 2018). A key limitation is that LEFM models only define whether or not a crack will propagate, so *ad hoc* criteria are often necessary to determine how much and in which direction the crack will propagate, especially under mixed‐mode conditions (Francfort & Marigo, 1998). As noted by Yu et al. (2017), simulating crevasse propagation and calving using LEFM models in combination with Stokes‐based ice flow models requires cumbersome adaptive meshing algorithms and small mesh sizes (∼2 m at the crevasse tip), which makes it computationally unattractive.

An alternative approach to the zero stress and LEFM models for parameterizing ice shelf fracture is to implement continuum damage mechanics, wherein the evolution of the damage variable describes the nucleation and accumulation of microcracks and influences deformation (or flow) by weakening the material response (Lemaitre, 1992). Owing to the visco‐elasto‐plastic nature of ice, the creep damage model (Murakami, 1983; Murakami & Ohno, 1980; Murakami et al., 1988) was adopted and calibrated based on laboratory data (Duddu & Waisman, 2012; Pralong & Funk, 2005; Pralong et al., 2006) for modeling time‐dependent fracture. This damage model may be implemented in isotropic or anisotropic form; however, anisotropic damage is likely more consistent with patterned fractures observed on ice shelves, which are typically oriented perpendicular to the flow direction. While it has only been tested at the scale of individual crevasses and in isotropic form, the creep damage model was able to reasonably simulate two calving events in the Swiss Alps within a 2‐D full‐Stokes formulation (Pralong & Funk, 2005). Further progress with the isotropic creep damage model at similar spatial scales has included additional calibration for temperature dependence (Duddu & Waisman, 2012), nonlocal formulations (Duddu & Waisman, 2013; Duddu et al., 2013; Jiménez et al., 2017; Londono et al., 2017), and a modification to incorporate the effects of water pressure (Duddu et al., 2020; Mobasher et al., 2016). The main drawback of the creep damage model is that damage evolution is not defined based on strain energy release rate or the work per unit fracture surface area; this can be addressed by extending a phase field damage formulation for elastic media (Sun et al., 2021) to viscoplastic media. To our knowledge, only one study has conceptualized parameterizing the creep damage model for application into SSA simulations of large‐scale ice flow (Keller & Hutter, 2014). This study proposed updating the isotropic creep damage field in three‐dimension (3‐D) using parameterized Cauchy stress, and vertically averaging a 3‐D damage‐modified viscosity parameter for implementation into the 2‐D SSA solution. However, this parameterization remains untested, potentially due to the lack of a robust numerical framework that can handle ice flow and fracture evolution in a computationally feasible manner.

The overarching goal of this study is to implement the SSA creep damage parameterization within a robust numerical framework that can represent the entire progression of ice shelf fracture, from initiation and evolution of subcritical damage to propagation of sharp rifts and calving of tabular icebergs. Here, we extend the SSA parameterization of Keller and Hutter (2014) to include an anisotropic creep damage variable, and construct a supporting material point method that minimizes error and maximizes efficiency so that it may be applied effectively within large‐scale ice flow simulations. We adapt several schemes within this framework to improve model performance and physical consistency, including extension of the damage variable to nonlocal form, adaptive time‐stepping based on damage accumulation, brittle rupture criteria, and numerical treatment once maximum damage is reached. The damage model is implemented within our generalized interpolation material point method (GIMPM) code, which is a hybrid Eulerian‐Lagrangian particle variation of the finite element method (Huth et al., 2021). Traditional Eulerian ice flow models are subject to artificial diffusion when advecting the damage field (e.g., Albrecht & Levermann, 2014; Borstad et al., 2016), whereas this error is avoided when using our GIMPM‐SSA model, thereby allowing sharpness of cracks to be preserved regardless of flow. Additionally, the GIMPM‐SSA model increases the computational efficiency of advecting the 3‐D damage field, or any other 3‐D field such as temperature.

We test the SSA creep damage model on an idealized marine ice sheet system (Asay‐Davis et al., 2016) to demonstrate that it can capture all damage growth from initial accumulation to sharp rifting and tabular calving, and to explore parameter sensitivity. Additionally, we compare the performance of our model with two previously proposed crevasse‐depth‐based damage models (Bassis & Ma, 2015; Sun et al., 2017) by extending them from isotropic to anisotropic form. The outline of this study is as follows: in Section 2, we summarize the governing equations, including the SSA and damage parameterization; in Section 3, we detail the implementation of the damage model; in Section 4, we present the idealized ice sheet experiments; in Section 5, we discuss the results and potential future developments and applications; and in Section 6, we offer concluding remarks. As supporting information, we provide some additional simulation results and details of the crevasse‐depth‐based damage models of Bassis and Ma (2015) and Sun et al. (2017).

## 2 Governing Equations

We begin this section by briefly reviewing the SSA equations. Then, we present the creep damage model and its parameterization for the SSA. We use a mix of tensorial and indicial notation as needed for conciseness and clarity. Vectors are donated as $a=a_{i}{\hat{e}}_{i}$, where the indicial notation of the right‐hand side is framed within a Cartesian coordinate system $\left(x_{1},x_{2},x_{3}\right)=\left(x,y,z\right)$, i are the spatial indices and ${\hat{e}}_{i}$ are the orthonormal basis vectors. Second‐order tensors are similarly denoted as $A=\;A_{ij}{\hat{e}}_{i}⊗{\hat{e}}_{j}$, where ⊗ is the dyadic product of the Cartesian base vectors. We adopt Einstein's convention of summation that repeated indices imply summation. Principal values of A are written as $\langle A_{i}\rangle$, where in this case, index i indicates principal components rather than Cartesian directions. Variables at time step m are indicated using the superscript $A^{m}$. Constants used for the damage model are listed in Table 1, and all other constants are given in Table S1.

**Table 1:** Creep Damage Parameters Used in the Experiments

Parameter	Value	Reference
$B^{*}$	5.23 × 10^−7^ MPa^−*r*^ s^−1^	Duddu and Waisman (2012)
*r*	0.43	Pralong and Funk (2005)
$k^{*}$	4	Keller and Hutter (2014)
*α*	0.21	Pralong and Funk (2005)
*β*	0.63	Pralong and Funk (2005)
λ	0.16	Pralong and Funk (2005)
γ	0, 0.5, or 1	−
$\sigma_{\text{th}}$	0.12 MPa	−
$D_{\text{cr}}$	0.6	Duddu and Waisman (2013)
$D_{\text{max}}$	0.99	−
${\bar{D}}_{\text{cr}}$	0.8	−
${\bar{D}}_{\text{max}}$	0.9	−
${\bar{dD}}_{\text{max}}$	0.075	−
$\delta_{1}$	1.8	Ling et al. (2000)
$\delta_{2}$	0.05	Ling et al. (2000)
$\delta_{3}$	0.9	−
$l_{c}$	1 or 2 km	−

### 2.1 Shallow Shelf Approximation

Ice streams and ice shelves have little or no basal drag, so vertical shear is negligible. Consequently, horizontal velocities and the corresponding strain‐rates can be assumed constant with depth. Assuming that vertical normal stress is equal to the overburden pressure, excluding vertical shear components from the incompressible Stokes equations, and vertically integrating yields the 2‐D SSA, or SSA (Huth et al., 2021; MacAyeal, 1989; Weis, 2001; Section 2.1)

(1) $$\frac{\partial T_{ij}}{\partial x_{j}}+\;{\left(\tau_{b}\right)}_{i}=\;\rho gH\frac{\partial s}{\partial x_{i}}\;,$$

where i ranges over {1,2} to indicate the horizontal $x_{1}-x_{2}$ plane, *ρ* is the ice density, *g* is the acceleration due to gravity, *H* is the ice thickness, *s* is the surface height above sea level, $\tau_{b,i}$ are the horizontal components of the shear stress vector for basal drag, and $T_{ij}$ is a vertically integrated stress tensor

(2) $$T_{ij}=2\bar{\eta }H\left({\dot{\varepsilon }}_{ij}\;+\left({\dot{\varepsilon }}_{11}\;+{\dot{\varepsilon }}_{22}\right)\delta_{ij}\right)\;.$$

In Equation 2, ${\dot{\varepsilon }}_{ij}$ is the strain rate tensor, $\;\delta_{ij}$ is the Kronecker delta, and $\bar{\eta }$ is the depth‐averaged isotropic viscosity

(3) $$\bar{\eta }=\frac{1}{2}\bar{B}{\dot{\varepsilon }}_{e}^{\frac{1-n}{n}}\;,$$

where, ${\dot{\varepsilon }}_{e}=\sqrt{\varepsilon_{ij}\varepsilon_{ji}/2}$ is the equivalent strain rate, *n* is the Glen's Law exponent set to *n* = 3, and $\bar{B}$ is the depth‐averaged isotropic stiffness parameter. At the ice‐ocean boundary (or ice front), the seawater pressure is applied using a depth‐integrated Neumann boundary condition as

(4) $$\overset{s}{\underset{b}{\int }}\sigma_{ij}{\hat{n}}_{j}\;dz=-\frac{1}{2}\rho_{w}gb^{2}{\hat{n}}_{i}\;,$$

where σ is the Cauchy stress, $\hat{n}$ is the unit (outward) normal to the ice front, $\rho_{w}$ is the seawater density, and b is the elevation of the ice draft (Morland & Zainuddin, 1987). The SSA is solved for the in‐plane velocity components $\left(v_{1},v_{2}\right)$ of the ice shelf/stream by reformulating Equations 1 and 2 in terms of the velocity gradients derived from the strain rate tensor ${\dot{\varepsilon }}_{ij}=\frac{1}{2}\left(\frac{\partial v_{i}}{\partial x_{j}}+\frac{\partial v_{j}}{\partial x_{i}}\right)$. Along with momentum balance equations in Equation 1, the ice thickness is updated based on surface mass balance for a column of ice as

(5) $$\frac{\partial H}{\partial t}\;=\left(\dot{a}\;-\;\nabla \cdot vH\right)\;,$$

where $\dot{a}$ (m a^−1^) is the sum of the basal and surface accumulation rates (Huth et al., 2021). In our GIMPM‐SSA framework (Huth et al., 2021), ice velocity is determined using the Eulerian description, whereas ice thickness is explicitly updated at material points or particles using the Lagrangian description. Thus, it can be classified as a hybrid Eulerian‐Lagrangian method.

### 2.2 Physical Notion of Continuum Damage

We implement the anisotropic creep damage model originally proposed by Murakami (1983), Murakami and Ohno (1980), and Murakami et al. (1988) for polycrystalline metals. Pralong and Funk (2005) first calibrated this model for glacier ice and discussed the thermodynamic considerations in Pralong et al. (2006). Damage is represented as a real‐valued, symmetric second‐order 3‐D tensor, D, so that anisotropy is restricted to an orthotropic description where damage is tracked on three mutually perpendicular planes. The damage tensor has three real principal values, $\langle D_{i}\rangle$, each representing the ratio of the area of cracks or voids to the originally undamaged area along the principal plane with a normal corresponding to principal direction *i* (Duddu & Waisman, 2013; Murakami, 1983). This physical or geometric interpretation is valid under isotropic $(\langle D_{1}\rangle =\;\langle D_{2}\rangle =\;\langle D_{3}\rangle )\;$ and orthotropic damage (Qi & Bertram, 1999). Each principal damage component is bounded by $\left(0\;\leq \langle D_{i}\rangle \leq D_{\text{max}}\right)$, where a material point is undamaged if all $\langle D_{i}\rangle =0$ and fully damaged if any $\langle D_{i}\rangle =D_{\text{max}}$. Setting $D_{\text{max}}$ to the maximum possible value of unity corresponds to complete loss of strength, though numerically, $D_{\text{max}}$ must be set less than unity to prevent the SSA from becoming an ill‐posed problem. Given the free‐slip conditions that allow for the vertical plug‐flow regime of the SSA, we assume that the damage tensor is oriented so that one principal component, which we denote as $\langle D_{3}\rangle$, always aligns with the vertical $x_{3}$ axis $\left(\langle D_{3}\rangle =\;D_{33}\right)$. The other two principal axis lie in the horizontal $x_{1}-x_{2}$ plane, where we always ensure $\langle D_{1}\rangle \;\geq \langle D_{2}\rangle$. Because vertical shear stress components are neglected in the SSA, the orthotropic damage tensor has only four non‐zero components $D_{11}$,$\;D_{22}$, $D_{33}$, and $D_{12}$ that need to be determined.

The damage evolution function and incorporation of the damage tensor into the SSA rely on the principle of strain equivalence (Lemaitre, 1971; Lemaitre & Chaboche, 1978). This principle states that strain is identical for a damaged state under the applied stress, $\sigma_{ij}$ (force per unit ice area, including void spaces), as for its undamaged state under the effective stress, ${\tilde{\sigma }}_{ij}$ (force per unit ice area, excluding voids). A linear transformation between the two stress spaces that ensures the symmetry of the effective stress tensor can be written as (Pralong & Funk, 2005)

(6) $$\tilde{\sigma }\;=\;\frac{1}{2}[{\left(I-D\right)}^{-1}\sigma +\sigma {\left(I-D\right)}^{-1}]\;,$$

where I is the second‐order identity tensor, that is, the matrix form of the Kronecker delta. An effective strain‐rate is used to incorporate damage into the constitutive relation and calculate the applied stress, and takes the form

(7) $$\tilde{\dot{\varepsilon }}\;=\;\frac{1}{2}{\left[\left(I-D)\dot{\varepsilon }+\dot{\varepsilon }(I-D\right)\right]}^{D}\;$$

where the superscript “D” refers to the deviatoric part of the second order tensor, which is obtained by subtracting the mean of the diagonal components from each diagonal component of the tensor.

### 2.3 Damage Evolution Function

The creep damage evolution function is expressed in rate form. While some SSA damage models assume damage updates instantaneously with the stress field in a brittle manner (e.g., Sun et al., 2017), a rate form is consistent with laboratory experiments on creep failure of ice (Duddu & Waisman, 2012). In the Lagrangian framework, we express the material derivative of the second‐order creep damage tensor as the Jaumann derivative (Pralong & Funk, 2005)

(8) $$\dot{D}=\frac{\partial D}{\partial t}=f+\mathrm{WD}-\mathrm{DW}\;,$$

where *t* is the time, W is the spin tensor $W_{ij}=\frac{1}{2}\left(\frac{\partial v_{i}}{\partial x_{j}}-\frac{\partial v_{j}}{\partial x_{i}}\right)$, and f is the dynamic damage evolution function given as (Murakami et al., 1988)

(9) $$f=B^{*}{\langle \langle \chi -\;\sigma_{\text{th}}\rangle \rangle }^{r}{\left\{\text{Tr}\left[\;{\left(I-D\right)}^{-1}\cdot \left({\hat{\xi }}^{\left(1\right)}⊗{\hat{\xi }}^{\left(1\right)}\right)\right]\;\right\}}^{k^{*}}\left[(1-\gamma )I+\gamma {\hat{\xi }}^{\left(1\right)}⊗{\hat{\xi }}^{\left(1\right)}\right]\;,$$

(10) $$\chi =\alpha \langle {\tilde{\sigma }}_{1}\rangle +\;\beta \sqrt{\frac{3}{2}\text{Tr}[{\left({\tilde{\sigma }}^{D}\right)}^{2}]}+(1-\alpha -\beta )\text{Tr}[\tilde{\sigma \;}]\;.$$

In Equation 9, $B^{*}$, r, and $k^{*}$ are the creep damage parameters (listed in Table 1), Tr[⋅] denotes the trace operator, and χ is the Hayhurst stress defined in Equation 10, which is an equivalent stress measure (Hayhurst, 1972). The Hayhurst stress is a weighted combination of the maximum (most tensile, where tension is positive) effective principal stress (weighted by α), the effective von Mises stress (weighted by β), and the trace of the effective stress (weighted by λ = 1−α−β), which is three times the volumetric stress. The Hayhurst weights must satisfy the condition

(11) 0≤α,β,λ≤1.

Here, we take α = 0.21, β = 0.63, and λ = 0.16 as previously calibrated from laboratory data (Pralong & Funk, 2005). The first term in Equation 9 determines the damage evolution rate based on the Hayhurst criterion and $\sigma_{\text{th}}$, an assumed stress threshold that restricts damage evolution to where $\;\chi \;>\;\sigma_{\text{th}}$. The Macaulay brackets 〈〈⋅〉〉 are defined as

(12) $$\langle \langle x\rangle \rangle =\left\{\begin{array}{l} x,\;\text{if}\;x\geq 0\\ 0,\;\text{if}\;x<0\; \end{array}\right.\;.$$

In the second and third terms of Equation 9, ${\hat{\xi }}^{\left(1\right)}$ is the eigenvector corresponding to the maximum effective principal stress, $\langle {\tilde{\sigma }}_{1}\rangle$, which we always assume lies within the horizontal $x_{1}-x_{2}\;$ plane to be consistent with crevasse formation along vertical planes. Operator Tr[⋅] denotes the trace. Although the exponent $k^{*}$ has been calibrated based on laboratory experimental data to be a function of the Cauchy stress (e.g., Duddu & Waisman, 2012; Pralong & Funk, 2005), we take it as a constant here for simplicity. The second term of Equation 9 accounts for the tendency of the damage accumulation rate to increase proportionately with the amount of pre‐existing damage along the damage plane. The third term sets the level of anisotropy in damage accumulation according to the anisotropy weighting parameter γ, which can be set between zero (purely isotropic with damage accumulating on all principal planes equally) and one (purely anisotropic with damage accumulating only on the principal plane normal to the ${\hat{\xi }}^{\left(1\right)}$ direction). According to Equation 9, if D and $\tilde{\sigma }$ are always coaxial, the relationship between the principal components of the damage rate is controlled by the anisotropy parameter as

(13) $$\langle {\dot{D}}_{2}\rangle =\langle {\dot{D}}_{3}\rangle =\left(1-\gamma \right)\langle {\dot{D}}_{1}\rangle \;.$$

Any misalignment between D and $\tilde{\sigma }$ will cause damage accumulation to become more weighted toward $\langle D_{2}\rangle$ at the expense of $\langle D_{1}\rangle$. Misalignment can occur, for example, as a rift develops and causes the orientations of principal stresses to change downstream. Note that growth of $\langle D_{3}\rangle$ is probably not physically motivated because it corresponds to development of horizontal crevassing, rather than vertical crevassing. From Equations 9 and 13, and given our assumption that the maximum effective principal stress always lies within the horizontal $x_{1}-x_{2}\;$ plane, it is evident that damage will accumulate on $\langle D_{3}\rangle$ if γ<1, but not if damage is fully anisotropic (γ=1). We test sensitivity to γ in Section 4.2.

### 2.4 Parameterization of Creep Damage for the SSA

While the SSA is 2‐D, we must determine the effective Cauchy stress field in 3‐D in order to evolve creep damage using Equations (8), (10). Damage can then be vertically averaged for incorporation into the next SSA solution step (Section 3.3). Following the SSA creep damage parameterization previously proposed by Keller and Hutter (2014), we calculate the effective Cauchy stresses at vertical coordinate z according to the deviatoric stresses, a parameterized pressure, and the existing damage. The deviatoric stresses are obtained at vertical coordinate z using the nonlinearly viscous constitutive relation for ice flow (Glen, 1955)

(14) $$\sigma^{D}\left(z\right)=2\eta \left({\dot{\varepsilon }}_{e},z\right)\tilde{\dot{\varepsilon }}\left(z\right)\;,$$

where $\tilde{\dot{\varepsilon }}$ is determined according to Equation (8), (10) using the 2‐D strain‐rates from the SSA solution and the local 3‐D damage. Isotropic viscosity $\eta =\frac{1}{2}B\left[T^{*}\left(z\right)\right]{\dot{\varepsilon }}_{e}^{\frac{1-n}{n}}$ is the depth‐dependent form of Equation (8), (10), where 3‐D stiffness parameter, B, is dependent on temperature, $T^{*}\left(z\right)$, which may be depth‐varying. The ice pressure used to derive the SSA is approximated using the hydrostatic assumption (Greve & Blatter, 2009)

(15) $$p_{i}\left(z\right)=\rho g\left(s-z\right)-\sigma_{11}^{D}\left(z\right)-\sigma_{22}^{D}\left(z\right).$$

However, while this pressure is appropriate for vertically integrated viscous ice flow, it should not be used for 3‐D damage evolution because it does not account for the opposing pressure of seawater that penetrates into basal crevasses in the ice shelf. This seawater pressure reduces the local ice pressure compared to Equation (8), (10), promoting basal crevasse propagation. Following Keller and Hutter (2014), we use the effective pressure

(16) $$p_{\text{eff}}=p_{i}-p_{w},$$

where $p_{w}$ is the seawater pressure

(17) $$p_{w}\left(z\right)=\left\{\begin{array}{l} 0,\;\text{if}\;z\geq z_{\text{sl}}\\ \rho_{w}g\left(z_{\text{sl}}-z\right),\;\text{if}\;z<z_{\text{sl}} \end{array}\right.\;.$$

with $z_{\text{sl}}$ denoting the sea level elevation, which we set to zero. Note that this effective pressure is only used for damage evolution; whereas, we use the ice pressure defined in Equation (8), (10) in our formulation of the SSA.

With these definitions of deviatoric stress and effective pressure, Cauchy stress is calculated as $\sigma_{ij}\left(z\right)=\;\sigma_{ij}^{D}\left(z\right)-p_{\text{eff}}\left(z\right)\delta_{ij}$. It may appear that this stress could then be converted to the needed effective stress using Equation (8), (10). However, Keller and Hutter (2014) suggest that because the compressive ice overburden dominates the volumetric stress in the SSA, only the deviatoric stresses should be affected by damage, so that Equation (8), (10) is invalid. Therefore, the effective stress is instead given as ${\tilde{\sigma }}_{ij}=\;{\tilde{\sigma }}_{ij}^{D}-p_{\text{eff}}\delta_{ij}$, where we define the effective deviatoric stress as (Pralong et al., 2006)

(18) $${\tilde{\sigma \;}}^{D}=\;\frac{1}{2}{\left[{\left(I-D\right)}^{-1}\sigma^{D}+\sigma^{D}{\left(I-D\right)}^{-1}\right]}^{D}\;.$$

The Hayhurst criterion Equation (8), (10) is then re‐expressed for the SSA as

(19) $$\chi_{\text{SSA}}=\alpha \left(\;\langle {\tilde{\sigma }}_{1}^{D}\rangle -p_{\text{eff}}\;\right)+\;\beta \sqrt{\frac{3}{2}\text{Tr}\left[{\left({\tilde{\sigma }}^{D}\right)}^{2}\right]}+\lambda \left(-3p_{\text{eff}}\right)\;.$$

As emphasized by Keller and Hutter (2014), these definitions of effective pressure and effective stress are only parameterizations, so different values for the Hayhurst weights and the stress threshold from those calibrated in Pralong and Funk (2005) may be appropriate as well. Alternative pressure and stress parameterizations may also be valid, which we discuss further in Section 5.2.

## 3 Implementation

We start this section by discussing the GIMPM‐SSA framework, including how damage is implemented within it and its advantages concerning accuracy and efficiency of the ice flow and damage solutions. We then present the solution for the local 3‐D damage increment, and explain how it can be used to set an adaptive time step and diffused over a characteristic length scale to calculate a nonlocal damage increment. Furthermore, we describe a brittle rupture criterion, the depth‐averaging of the 3‐D damage field, and our current treatment of fully damaged material points (rifts). Lastly, we detail incorporation of the depth‐averaged damage variable into the SSA solution.

### 3.1 Generalized Interpolation Material Point Method (GIMPM)

If using mesh‐based numerical methods, then artificial diffusion errors may arise during advection of the damage variable, which smear sharp edges and make critical features such as rifts difficult to capture. This diffusion is inherent to purely Eulerian advection schemes, where the mesh is not moved with the computed velocity field, and can also arise when working in a Lagrangian frame (moving‐mesh) due to frequent remeshing that may be required when modeling large‐deformation processes like ice shelf flow. While our creep damage model may be adopted for any flow‐modeling framework, we implement it here within our GIMPM‐SSA code to avoid these diffusion errors (Huth et al., 2021). The GIMPM (Bardenhagen & Kober, 2004) is one of several material point methods, which all share the same basic procedure. In the GIMPM, a set of material points (or particles) provides a Lagrangian description of the material domain and holds all dynamic variables. The momentum equations are solved on a background grid in a similar manner to the finite element method, but with the material points serving as moving integration points. The grid solution is then used to update material point quantities such as position, velocity, and area, as well as material point history variables. Here, the history variables are ice thickness and damage. These updates are performed in a Lagrangian frame, which ensures that all fields advect without diffusion errors and enables tracking of the ice front and grounding line at sub‐grid accuracy. The primary difference between the various material point methods concerns the shape functions used to map between material points and the grid. The most accurate variants use C^1^ continuous shape functions to ensure smooth transfers of stiffness as material points move between grid cells, and in the GIMPM, such shape functions are assembled by convolving linear grid functions with characteristic functions associated with each material point (see Huth et al., 2021 for details).

Within the GIMPM‐SSA framework, we track damage and any other 3‐D fields, such as temperature, upon a series of vertical layers assigned to each material point. For mesh‐based methods, the vertical layers could be assigned to nodes or quadrature points instead. For the simulations in this study, we always maintain an even distribution of layers between the local ice base and surface elevations, which is possible because we do not incorporate mass balance processes such as surface and basal melt, or infill of crevasses with snow at the surface or marine ice at the base. Therefore, the only vertical motion of layers here is driven by changes in ice thickness from extension or compression, owing to the H(∇⋅v) term in Equation (8), (10). Furthermore, we do not account for necking processes (Bassis & Ma, 2015) or healing due to crack closure under compression (Pralong & Funk, 2005; Pralong et al., 2006). Modifying the creep damage model to account for the impacts of mass balance, necking, and healing is beyond the scope of this study. However, in Section 4.4, we test a damage model for comparison that does account for some of these processes (Bassis & Ma, 2015), and we discuss the potential for a combined approach between the models in Section 5.1.

### 3.2 Local 3‐D Damage Increment

The 3‐D damage updates take the form

(20) $$D^{m+1}=\;D^{m}+\Delta D^{m}\;,$$

where $\Delta D^{m}$ is the damage increment over a time step and may be expressed in local or nonlocal form. For each material point layer, the local damage increment, ΔlocDm, is found by integrating the damage evolution rate, ${\dot{D}}^{m}$, over the length of the time step Δt using the Runge‐Kutta‐Merson (RKM) method as detailed in Ling et al. (2000) and Zolochevsky et al. (2009). The RKM update allows higher accuracy and longer time steps than a forward Euler update. During the RKM scheme, an internal damage variable is continuously updated over a series of sub‐steps, whose sizes are optimized for speed and accuracy. The strain‐rate determined from the preceding SSA solution is unchanged during the RKM update. The damage rate is calculated by solving Equations (7), (8), (9) and (14), (15), (16), (17), (18), (19). At completion, the RKM routine returns the local damage, ΔlocDm+1, from which ΔlocDm can be calculated as ΔlocDm=Dlocm+1−Dm.

We stop damage accumulation on a layer once the maximum principal damage component reaches $D_{\text{max}}$, though further evolution via the spin terms in Equation (8), (10) is allowed. A damage component that reaches $D_{\text{max}}$ is considered ruptured, and can roughly be associated with the formation of macrocracks or crevasses, though we currently make no explicit assumptions concerning their width, spatial distribution, or potential influence on driving stress. However, our SSA damage model is probably most consistent to describe the propagation of isolated or widely spaced crevasses because we do not account for the shielding effects from damaged layers of neighboring material points. Stopping damage accumulation once $\langle D_{1}\rangle =D_{\text{max}}$ is a requirement of the current formulation of the damage model, which does not currently account for multi‐axial damage accumulation after rupture. Therefore, our model does not currently allow development of cross‐cutting crevasses, though we estimate their occurrence and influence on flow is typically minimal for ice shelves. However, multi‐axial damage accumulation before rupture, which may occur under biaxial tension, could possibly be accounted for by modifying the anisotropy parameter according to the relative magnitude of the two tensile principal effective stresses (Ganczarski & Skrzypek, 2001). This multi‐axial modification has yet to be verified for ice, and has minimal impact on the experiments presented here. Therefore, we present the results that did not use this modification.

We split the above solution for the 3‐D damage increments into two loops over the layers of a material point. The first loop is run from the bottom layer toward the top layer, and is exited if a layer is encountered with ΔlocDm=0 and $D^{m}$ = 0 for all components. If the first loop does not process all layers, a second loop from the surface toward the base is initiated with the same exit criterion. During the second loop, we assume damage is associated with surface crevassing, so we remove the seawater pressure term in the effective pressure in Equation (8), (10). A surface meltwater pressure term could be added, but we do not currently account for this. This two‐loop scheme assumes cracks will not initiate in the middle of the shelf, and consequently, we achieve a faster solution by avoiding processing layers that will remain undamaged.

### 3.3 Adaptive Time‐Stepping

The maximum change in vertically averaged local damage, ${\bar{dD}}_{\text{max}}$, of all material points is used to adjust the time step as needed for both the current and next computational cycle, with the goal of limiting the amount of damage allowed to accumulate each cycle to ensure accuracy, stability, and efficiency. Because the damage update can affect the current time step, it must begin each computational cycle. We define ${\bar{dD}}_{\text{max}}$ as

(21) dD¯max=max(〈D¯m+1loc〉−〈D¯m〉),

where “max” on the right hand side indicates the maximum value of all principal components, and vertical averaging of the damage variables takes the form

(22) $$\bar{D}=\frac{\int_{b}^{s}D\left(z\right)B\left[T^{*}\left(z\right)\right]dz}{\int_{b}^{s}B\left[T^{*}\left(z\right)\right]dz}\;,$$

The integrals are evaluated using the trapezoid rule. Note that since $B\left[T^{*}\left(z\right)\right]$ can vary with depth, it must be included in Equation (8), (10) alongside D(z) to properly capture the combined effect of damage and thermal softening on the depth‐averaged viscosity of ice (Keller & Hutter, 2014).

If ${\bar{dD}}_{\text{max}}\geq 0.075$, we decrease the current time step as $\Delta t^{m}=\;\Delta t^{m}/1.5$ and recalculate the local damage increments. This situation rarely occurs, but serves as a safeguard against rapidly increasing damage. If ${\bar{dD}}_{\max}<0.075$, the time step for the next computational cycle is set as

(23) $$\Delta t^{m+1}=\min\left(\delta_{1}\Delta t^{m}\;,\;\frac{\delta_{2}\Delta t^{m}}{{\bar{dD}}_{\text{max}}},\;\text{CFL}\right)\;,$$

where, we take $\delta_{1}=1.8$ and a $\delta_{2}$ of 0.05 (Ling et al., 2000), and $\text{CFL}=\delta_{3}/\text{max}\left(|\frac{v_{1}}{\Delta x_{1}}|+\left|\frac{v_{2}}{\Delta x_{2}}\right|\right)$ indicates the maximum time step that satisfies the Courant‐Friedrichs‐Lewy (CFL) condition with constant $\delta_{3}\leq 1$. Here, the time step is almost always restricted by damage rather than the CFL condition, and consequently, ${\bar{dD}}_{\text{max}}\approx \delta_{2}$ each computational cycle. The typical time increment varies based on the chosen damage parameters, but in all the simulations in this study, it is on the order of days for subcritical damage accumulation to hours during rapid rift propagation.

### 3.4 Nonlocal 3‐D Damage Increment

Implementing nonlocal damage is motivated by both physical and numerical considerations. Physically, the progressive accumulation of microcracks that damage mechanics describes is distributed over a characteristic length scale in quasi‐brittle materials like glacier ice (Bazant, 1986; Hall & Hayhurst, 1991). Numerically, local damage models suffer from directional mesh bias and mesh size sensitivity as damage localizes to single elements. We implement a nonlocal integral scheme (Duddu & Waisman, 2013), which diffuses the local damage increment between neighboring material points over the characteristic length scale. Note the difference between this intentional diffusion and the artificial diffusion that may arise using mesh‐based advection schemes: The nonlocal damage diffusion is physically based on observations of fracture in quasi‐brittle materials, whereas artificial diffusion is a numerical error that causes ice to lose damage unphysically over time.

Here, we apply the nonlocal scheme within each layer of neighboring material points. For example, local damage of the second layer of a material point is only reweighted according to the local damage of the second layer from surrounding material points, but not the layer above or below it. The nonlocal damage increment, $\Delta D^{m}\left(x^{m}\right)$, is calculated as

(24) ΔDm(xm)=∑j=1Nϕ(xm−x^jm)ΔlocDm(x^jm)∑j=1Nϕ(xm−x^jm),

where N is the number of material points, ${{\hat{x}}_{j}}^{m}$, positioned within a characteristic length, $l_{c}$, of $x^{m}$ at time step m. The weight function, ϕ is a Gaussian curve given as

(25) $$\phi \left(x^{m}-{{\hat{x}}_{j}}^{m}\right)=\;\text{exp}\left(-{\left(\frac{2∥x^{m}-{{\hat{x}}_{j}}^{m}∥}{l_{c}}\right)}^{2}\right)\;.\;$$

The nonlocal length, $l_{c}$, should reflect the size of the fracture process zone and should be set so that the number of neighboring material points, *j*, is large enough to alleviate grid dependence (Duddu & Waisman, 2013). We note that as an alternative to the nonlocal integral scheme presented here, an implicit‐gradient nonlocal scheme could be implemented, instead (Jiménez et al., 2017), which would require solving an equation on the mesh for each layer.

### 3.5 3‐D Damage Update

On each material point layer, the 3‐D damage tensor is updated from the damage increment according to Equation (8), (10). Afterward, a brittle rupture or failure criterion is enforced, where if the principal value $\langle D_{1}^{m+1}\rangle$ for a layer reaches a specified critical damage, $D_{\text{cr}}$, then it is set to $D_{\text{max}}$. The other two principal values $\langle D_{2}^{m+1}\rangle$ and $\langle D_{3}^{m+1}\rangle$ are also updated in a similar manner to Equation 13 as $\langle {D_{2}^{m+1}}_{\;}\rangle =\langle D_{3}^{m+1}\rangle =\left(1-\gamma \right)\langle D_{\text{max}}\rangle$, unless this update reduces their values. This rupture criterion accounts for the transition from subcritical damage, associated with the gradual nucleation and growth of microcracks and microvoids, to rapid critical crack growth, or rupture of a macrocrack, once crack density reaches a critical value. The value for the critical damage, $D_{\text{cr}}$, can be estimated from uniaxial tension data (Duddu & Waisman, 2012; Pralong & Funk, 2005). Values of $D_{\text{cr}}$ used in other ice damage models range from $D_{\text{cr}}=0.45$ (Duddu & Waisman, 2012) to 0.6 (Duddu & Waisman, 2013), and we set $D_{\text{cr}}$ to 0.6 throughout this study. Note that not all damage tensors on all layers of a material point are guaranteed to have the same orientation. Misalignments with depth can occur as damage initiates at different times and accumulates under varying stress fields over time. However, misalignment is minimal in the simulations presented here.

### 3.6 2‐D Damage Update and Rift Treatment

After the 3‐D damage update, the vertically averaged damage that will be implemented into the SSA, ${\bar{D}}^{m+1}$, is calculated according to Equation (8), (10). As was done for 3‐D damage, a 2‐D brittle rupture condition can be set by defining a vertically averaged critical damage, ${\bar{D}}_{\text{cr}}$, and maximum damage, ${\bar{D}}_{\text{max}}$. However, upon brittle rupture in 2‐D, we set all components of $\bar{D}$ to ${\bar{D}}_{\text{max}}$ rather than only the maximum principal component as in the 3‐D case. This 2‐D treatment is consistent with complete failure of the material point, or the formation of a rift. Larger values of ${\bar{D}}_{\text{max}}$ are associated with a faster rate of rift widening and greater downstream velocities, and we find values for ${\bar{D}}_{\text{max}}$ of approximately 0.85–0.9 produce well‐controlled and distinct rifts for the simulations presented here. Physically, setting a value of ${\bar{D}}_{\text{max}}$ less than unity can be interpreted as allowing some residual strength between the flanks of the rift, which can occur when rifts contain ice mélange that is structurally coherent enough to transmit stresses (Borstad et al., 2013; Larour et al., 2004b; Rignot & MacAyeal, 1998). A complete description of rift forces should include a boundary condition on the rift flank walls similar to that at the ice front (Equation (8), (10)), but which also accounts for the pressure of ice mélange and potential contact between rift flanks (Larour et al., 2014; Lipovsky, 2020). For simplicity, we do not explicitly implement such a boundary condition here; rather, its effect on the rift opening rate is simply parametrized by setting the value of ${\bar{D}}_{\text{max}}$ lower than unity. We discuss the potential for implementing more complex rift dynamics, including a rift wall boundary scheme, within the damage and GIMPM‐SSA framework in Section 5.2.

### 3.7 SSA Solution and Material Point Updates

Damage is incorporated into the SSA solution by replacing $\dot{\varepsilon }$ in (8), (10) with $\tilde{\dot{\varepsilon }}$, which is calculated from Equation (8), (10) using $\bar{D}$ as the damage variable. This substitution modifies the original SSA‐GIMPM discretization (see Huth et al., 2021), yielding the following element submatrices of the tangent matrix, K, that are computed by summing over material points:

(26) $$\begin{array}{l} K_{11IJ}\;:\;=\;\sum_{p=1}^{n_{p}}A_{p}\;{\bar{\eta }}_{p}H_{p}\;\left\{\frac{\partial S_{Jp}}{\partial x_{1}}[\;2\frac{\partial \phi_{Ip}}{\partial x_{1}}(2-D_{11}-D_{33})-\frac{\partial \phi_{Ip}}{\partial x_{2}}D_{12}]\;\right.\\ \;+\;\left.\frac{\partial S_{Jp}}{\partial x_{2}}\;[\;\frac{1}{2}\frac{\partial \phi_{Ip}}{\partial x_{2}}(2-D_{11}-D_{22})-\frac{\partial \phi_{Ip}}{\partial x_{1}}D_{12}]\;\;\right\}+\sum_{p=1}^{n_{p}}A_{p}{\hat{\beta }}_{p}\phi_{Ip}S_{Jp\;,}\\ K_{22IJ}\;:\;=\;\sum_{p=1}^{n_{p}}A_{p}\;{\bar{\eta }}_{p}H_{p}\left\{\;\frac{\partial S_{Jp}}{\partial x_{2}}[\;2\frac{\partial \phi_{Ip}}{\partial x_{2}}(2-D_{22}-D_{33})-\frac{\partial \phi_{Ip}}{\partial x_{1}}D_{12}]\;\right.\\ \;+\;\left.\frac{\partial S_{Jp}}{\partial x_{1}}\;[\;\frac{1}{2}\frac{\partial \phi_{Ip}}{\partial x_{1}}(2-D_{11}-D_{22})-\frac{\partial \phi_{Ip}}{\partial x_{2}}D_{12}]\;\;\right\}\;+\sum_{p=1}^{n_{p}}A_{p}{\hat{\beta }}_{p}\phi_{Ip}S_{Jp\;,}\\ K_{12IJ}\;:\;=\;\sum_{p=1}^{n_{p}}A_{p}\;{\bar{\eta }}_{p}H_{p}\left\{\;\frac{\partial S_{Jp}}{\partial x_{1}}[\;2\frac{\partial \phi_{Ip}}{\partial x_{2}}(1-D_{33})-\frac{\partial \phi_{Ip}}{\partial x_{1}}D_{12}]\;\right.\\ \;\;+\;\left.\frac{\partial S_{Jp}}{\partial x_{2}}\;[\;\frac{1}{2}\frac{\partial \phi_{Ip}}{\partial x_{1}}(2-D_{11}-D_{22})-\frac{\partial \phi_{Ip}}{\partial x_{2}}D_{12}]\;\right\},\\ K_{21IJ}\;:\;=\;\sum_{p=1}^{n_{p}}A_{p}\;{\bar{\eta }}_{p}H_{p}\left\{\;\frac{\partial S_{Jp}}{\partial x_{2}}[\;2\frac{\partial \phi_{Ip}}{\partial x_{1}}(1-D_{33})-\frac{\partial \phi_{Ip}}{\partial x_{2}}D_{12}]\;\right.\\ \;\;+\;\left.\frac{\partial S_{Jp}}{\partial x_{1}}\;[\;\frac{1}{2}\frac{\partial \phi_{Ip}}{\partial x_{2}}(2-D_{11}-D_{22})-\frac{\partial \phi_{Ip}}{\partial x_{1}}D_{12}]\;\right\}, \end{array}$$

In Equation (8), (10), material point parameters are indicated with the subscript p, where $A_{p}$ is the material point area, $\;{\hat{\beta }}_{p}$ is the friction parameter, and $n_{p}$ is the number of material points in the element. The nodal indices are indicated with I and J. We adopt the same shorthand from Part I (Huth et al., 2021) to notate the evaluation of the linear ($\phi_{Ip}$) and GIMPM ($S_{Jp}$) shape functions at a material point, where $\phi_{Ip}=\phi_{I}\left(x_{p}\right)$ and $S_{Jp}=\;S_{J}\left(x_{p}\right)$. After the SSA is solved, the computational cycle for the GIMPM then continues as described in Part I (Huth et al., 2021), where the grid solution is used to update material point velocity, 2‐D position, areal domain, and thickness. We use the algorithm eXtended Particle In Cell of order *k* (XPIC(k)) to perform the velocity and position updates, an algorithm that eliminates potential noise or overdamping associated with simpler update schemes (Hammerquist & Nairn, 2017). In agreement with a previous damage study (Nairn et al., 2017), we find that taking *k* = 5 yields sharp and stable crack propagation. Because each layer of a material point has the same horizontal velocity, updating the 2‐D position of the material points automatically accounts for advection of any 3‐D field, such as damage. Therefore, 3‐D advection is essentially computationally free in the GIMPM‐SSA framework. Conversely, using mesh‐based Eulerian methods for advection would require solving a 3‐D matrix equation for each damage component. Therefore, the Eulerian approach would be much more expensive than the GIMPM‐SSA framework; in addition, Eulerian advection schemes would suffer from artificial numerical diffusion.

## 4 Idealized Test Case: MISMIP+

We carry out three experiments to test the SSA creep damage model under different tunings and compare its performance to previously published SSA damage models. We begin each experiment from the undamaged steady state configuration from the Marine Ice Sheet Model Intercomparison Project (MISMIP+, Asay‐Davis et al., 2016), which we describe in Section 4.1. Then, we allow damage and ice flow to evolve over time. In Section 4.2, we report results for the creep damage model. We perform sensitivity tests for the anisotropy parameter, mesh resolution, the nonlocal length scale, and the impact of an isothermal versus linear temperature profile. For comparison, we test a crevasse‐tracking damage model (Sun et al., 2017) in Section 4.3, where crevasse depths are calculated using the “zero‐stress” criterion (Nye, 1957). We conduct further tests with the zero‐stress damage model in Section 4.4, but where we modify the model to also account for the effects on damage from necking and mass balance (Bassis & Ma, 2015).

### 4.1 MISMIP+

**Figure 1:**
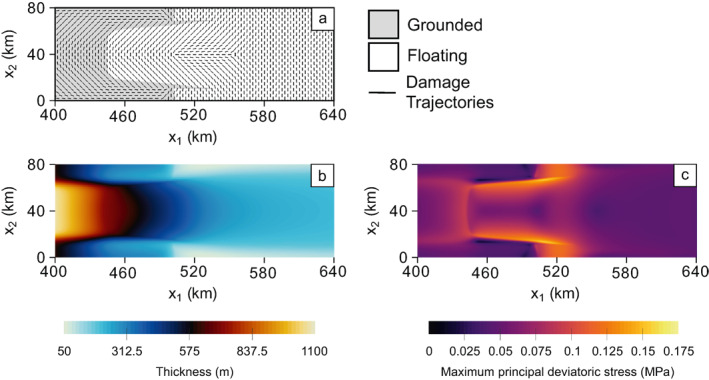
The Marine Ice Sheet Model Intercomparison Project (MISMIP+) steady‐state configuration near the grounding line. Panel (a) shows grounding line configuration and initial anisotropic damage trajectories. The trajectories correspond to the plane along which $\langle {\bar{D}}_{1}\rangle$ accumulates, and can be interpreted as crevasse patterns. The thickness field is given in (b) and the maximum principal deviatoric stress field is given in (c).

The MISMIP+ geometry is rectangular. In the longitudinal direction, the domain spans from an ice divide at $x_{1}=0$ km to an ice front at $x_{1}=640$ km. We do not allow the position of this ice front to evolve over time. The lateral boundaries span from $x_{2}=0$ to $x_{2}=80$ km, and the entire system has a plane of symmetry about $x_{2}=40$ km. The right side of the rectangular domain is an ice front, where the Neumann boundary condition Equation (8), (10) is applied. Normal velocities are set to zero (i.e., zero inflow/outflow, or roller boundary conditions) at all other boundaries. The bedrock topography is a U‐shaped submarine trough. Detail of the steady‐state grounding configuration is shown in the gray shading of Figure 1a, with the steady‐state thickness and maximum principal deviatoric stress fields given in Figure 1b and Figure 1c, respectively. At the most retreated section of the steady‐state grounding line ($x_{1}\;∼\;450$ km), the bed has a retrograde slope. The higher sidewalls of the bedrock trough result in thin, lateral protrusions of grounded ice that define the maximum longitudinal extent of the grounding line at $x_{1}\;∼\;537$ km. All floating ice upstream of this point constitutes a laterally supported shelf ice, whereas all ice downstream constitutes an unsupported floating ice tongue. The trajectories overlaying Figure 1a correspond to the second principal component of anisotropic damage at the first time step. These trajectories may be interpreted as crevasse patterns, that is, the planes parallel to the crevasse length, where the crevasse opens in the perpendicular $\langle {\bar{D}}_{1}\rangle$ direction.

Starting from a thin slab of ice defined over the domain, we grew the system to steady state using the given MISMIP+ ice flow parameters and accumulation rate and a modified Coulomb law for friction (Gagliardini et al., 2007; Leguy et al., 2014; Schoof, 2005). For this spin‐up procedure, we use the SSA and thickness evolution solvers in the finite element software Elmer/Ice (Gagliardini et al., 2013). Without the damage model, the GIMPM‐SSA model can hold the grounding line at its steady‐state position for at least 100 years if no melt rate is assigned, satisfying the MISMIP+ Ice0 control experiment (Huth et al., 2021). Unless otherwise specified, we use a structured rectangular mesh/grid with a resolution of 0.5 km and initiate nine regularly spaced material points within each grid cell.

### 4.2 SSA Creep Damage Simulations

We test our SSA creep damage model using the nonlocal integral formulation with the parameters given in Table 1, where α, β, and r, assume the values calibrated by Pralong and Funk (2005). We initially specify that the ice shelf is isothermal, so that the stiffness parameter, B, does not vary with depth, and we set a stress threshold of $\sigma_{\text{th}}=0.12$ MPa. We set a nonlocal length scale of $l_{c}=1$ km, which roughly corresponds to the size of the fracture process zone in the horizontal plane, which we estimate from clusters of seismicity detected around a propagating rift on Amery Ice Shelf (Bassis et al., 2007). For our initial creep damage experiment, we test three different levels of damage anisotropy: γ=0, γ=0.5, and γ=1, which correspond to fully isotropic, evenly mixed isotropic/anisotropic, and fully anisotropic damage, respectively. Each simulation eventually results in tabular calving, at which point we end the simulation. We report results for the 2‐D vertically integrated maximum principal damage.

#### 4.2.1 Initial Damage Accumulation

**Figure 2:**
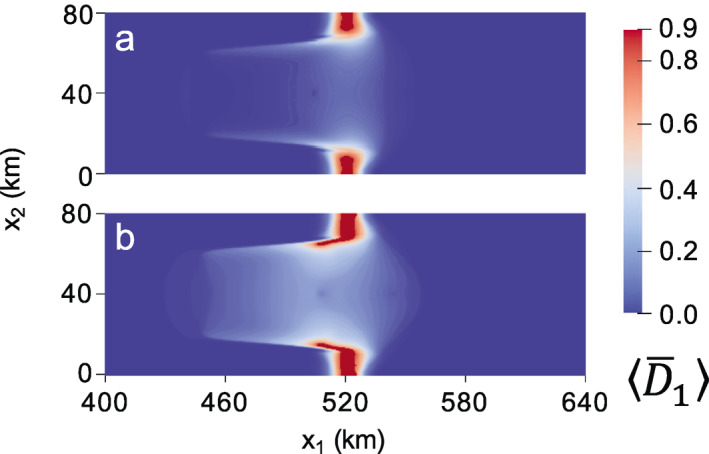
Maximum principal damage field for the fully anisotropic (γ=1) creep damage simulation at (a) 0.06 and (b) 0.2 years. Material points with $\langle {\bar{D}}_{1}\rangle ={\bar{D}}_{\text{max}}=0.9$ correspond to rifts.

For all simulations, damage accumulation is minimal for interior grounded ice because deviatoric stresses are low and wherever ice basal elevation is higher than it would be in floatation, the seawater pressure is less able to counter ice overburden in the effective pressure (Equation (8), (10)). Downstream portions of the ice tongue also accumulate minimal damage, as stresses are low. Therefore, we only report results near the grounding line, where damage is greatest. Figure 2 shows the early evolution of the principal damage field $\langle {\bar{D}}_{1}\rangle$ for the fully anisotropic case at (a) 0.06 years and (b) 0.2 years. Nearly identical early damage patterns develop for the other values of γ tested (see Figures S1a and S2a for the isotropic and mixed isotropic/anisotropic cases, respectively). At 0.06 years in the fully anisotropic case, relatively strong damage accumulates along the ice shelf shear margins as expected, where stresses are large. However, the dominant damage accumulation occurs at $x_{1}\;∼\;520$ km, where rifts initiate from the lateral boundaries of domain as indicated where $\langle {\bar{D}}_{1}\rangle ={\bar{D}}_{\max}=0.9$. Note that this region is the thinnest region of the domain (Figure 1c), so ice overburden pressure is low. Consequently, this region has very large tensile stress in the along‐flow direction, and rifts initiate there first despite the shear margins of the ice shelf showing larger maximum deviatoric stresses in the initial steady‐state (Figure 1c). Also note that the lateral boundaries of the domain ($x_{2}=0$ and $x_{2}=80$ km) can be considered symmetry boundaries because the normal velocities are set to zero, so that the rifts can be considered to have initiated from the center of small ice shelves. While rifts typically initiate at grounded margins, rift initiation from the center of ice shelves has occurred, for example, at Pine Island Glacier in Antarctica (Jeong et al., 2016).

The configuration in Figure 2a is maintained until the grounded lateral protrusions weaken and thin enough to allow the rifts to propagate through ∼0.1 years later, at which point these regions also unground. The rifts propagate upstream following the elevated damage that previously developed along the ice shelf shear margins, as shown in Figure 2b at 0.2 years. As in Figure 2a, rifts for the lower‐anisotropy cases also propagate into a similar configuration, but with faster rates of propagation for lesser anisotropy. A comparable rift configuration develops in the fully isotropic case by ∼0.12 years and in the mixed isotropic/anisotropic case by ∼0.18 years (Figures S1b and S2b).

#### 4.2.2 Tabular Calving

**Figure 3:**
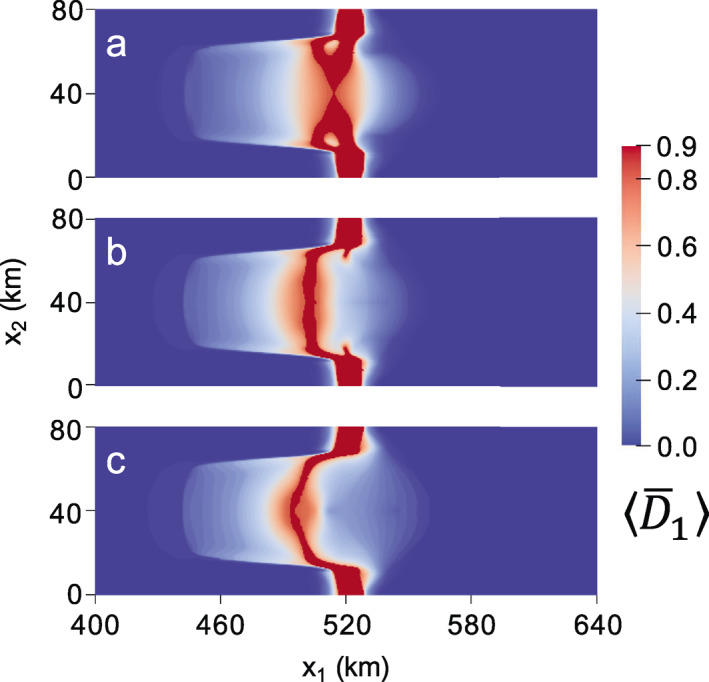
Maximum principal creep damage field at calving for: (a) isotropic (γ=0); (b) mixed isotropic‐anisotropic (γ=0.5); (c) fully anisotropic (γ=1) damage. The corresponding times to calving are (a) 0.165; (b) 0.272; (c) 0.486 years.

The rifting pattern in Figure 2b represents the final configuration before rifts propagate laterally across the domain to result in tabular calving. It is also the last configuration in which the spatial distribution of damage is similar for all values of γ. Figure 3 gives the final depth‐averaged principal damage field $\langle {\bar{D}}_{1}\rangle$ at calving. For the isotropic case (Figure 3a), the original rifts branch so that two points of calving occur; one branch originating from the upstream point of rifting reached in Figure S1b, and the other originating from a downstream position lateral to where the rift initiated at $x_{1}$ ∼ 520 km. This second branch also partially develops for the γ=0.5 case. However, for both the mixed isotropic/anisotropic (Figure 3b) and fully anisotropic (Figure 3c) cases, calving ultimately stems from the further upstream location.

Higher levels of anisotropy yield sharper and more arcuate rifts that are more characteristic of real ice shelves, and qualitatively describe “brittle” crack growth rather than “ductile” void growth under lower anisotropy. In Figure 3, higher anisotropy is also associated with slower rates of rift propagation, where the fully anisotropic case calves after 0.486 versus 0.165 years for the isotropic case. However, we emphasize that it is the anisotropy, not the speed of propagation, that allows the sharper rift and additional features to be captured. Rerunning the isotropic damage simulation with the damage rate factor $B^{*}$ that is four times smaller allows isotropic damage to evolve at a similar rate to the anisotropic case, but the damage pattern remains essentially unchanged. Similarly, lowering $\delta_{2}$ in Equation 23 so that less damage accumulates each time step has negligible effect. Lastly, we note that our choice of ${\bar{D}}_{\mathrm{cr}}$ = 0.8 was arbitrary, and effectively eliminating the rupture criterion by setting ${\bar{D}}_{\mathrm{cr}}$ = ${\bar{D}}_{\text{max}}$ still allows the same rift patterns to develop, but with a smoother transition in damage between ruptured and unruptured ice (not shown). However, the jump in damage induced by setting ${\bar{D}}_{\mathrm{cr}}$ lower than ${\bar{D}}_{\text{max}}$ yields more visually distinct rifting, and is likely physically justified because highly damaged shelf ice may experience vertical shear stresses not accounted for in the SSA (Bassis & Ma, 2015) that could contribute to full‐thickness brittle rupture.

In Figure 3, we observe that damage anisotropy strongly impacted rift behavior despite assuming damage isotropy post rupture with our simple scheme of representing rifts by setting all damage components of failed material points to ${\bar{D}}_{\text{max}}$. This suggests that the rift path is governed by anisotropic damage accumulation before full‐thickness rupture. The damage trajectories in Figure 1 show a clear arcuate pattern on the ice shelf that spans the lateral grounded margins, where the commonly observed pattern of en‐échelon crevassing is reproduced. Rift propagation more closely follows these trajectories with higher levels of damage anisotropy. Finally, we note that because none of these simulations lasted over six months, changes in the ice geometry were small and therefore had little impact on damage evolution. In this case, very similar damage evolution and rift propagation occurs even if material point ice thickness and positions are held constant over time.

#### 4.2.3 Sensitivity to Nonlocal Damage Length Scale

**Figure 4:**
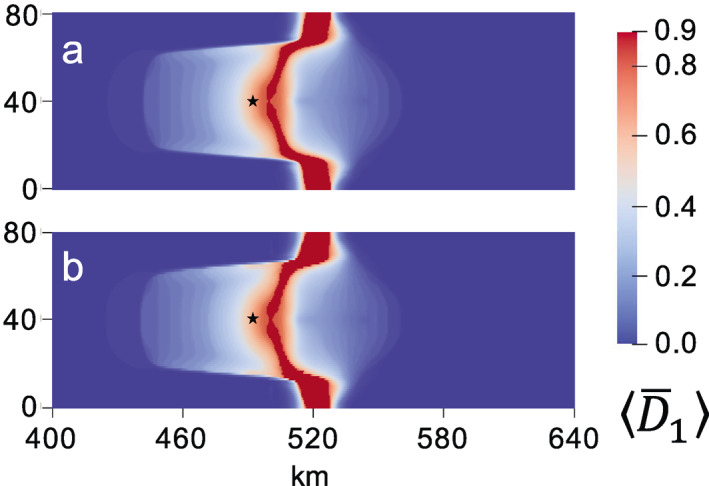
Maximum principal creep damage field at calving for fully anisotropic case (γ=1) when using a nonlocal length scale $l_{c}$ = 2 km and (a) 0.5‐km versus, (b) 1‐km grid resolution. Alleviation of grid dependence is evident in the similarity of damage patterns between the two simulations, as well as the comparable times to calving of (a) 0.493 and (b) 0.510 years. These rift patterns and calving times are also similar to those in Figure 3c, which uses a 0.5‐km grid and $l_{c}$ = 1 km. The most apparent difference is that rifting in the $l_{c}$ = 1 km case penetrates slightly farther upstream, as marked by the stars.

The choice of the nonlocal length, $l_{c}$, is important in determining the computational cost of simulations, because a larger $l_{c}$ allows larger element sizes to be used without grid bias. Ideally, $l_{c}$ should be three or four times the element size to guarantee that mesh dependence is alleviated (Duddu & Waisman, 2013; Jiménez et al., 2017). However, using $l_{c}$ = 1 km, which is twice the element size, appears to be sufficient in the above simulations; doubling $l_{c}$ to 2 km and rerunning the fully anisotropic case with 0.5 km grid resolution yields a similar rift path and time to calving (Figure 4a) as the 1‐km case (Figure 3c). To further confirm that grid dependence is alleviated, we rerun this $l_{c}$ = 2 km fully anisotropic case with 1‐km grid resolution. Again, a similar rift path and time to calving is realized (Figure 4b). The largest difference between simulations using $l_{c}$ = 2 km and $l_{c}$ = 1 km is that the latter produces rifts that penetrate slightly further upstream, as indicated by the stars in Figure 4. The insensitivity of the model response to the exact value of $l_{c}$ is encouraging given our rudimentary estimate of the size of the fracture process zone, which could theoretically vary in shape and size according to stress and damage. Alternative nonlocal integral formulations are available to modify the nonlocal zone according to these variables (e.g., Giry et al., 2011), but the observed insensitivity to $l_{c}$ likely obviates the need for these more complex nonlocal schemes.

#### 4.2.4 Effect of Temperature Gradient

**Figure 5:**
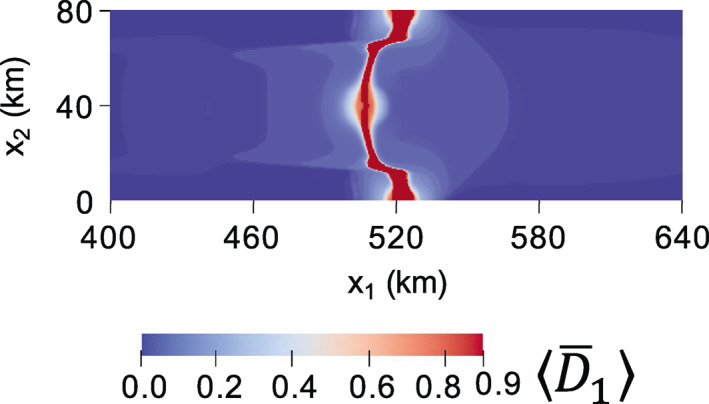
Maximum principal damage field at calving for fully anisotropic (γ=1) creep damage when using the linear temperature profile and $l_{c}$ = 1 km.

Our final test with the creep damage model highlights how vertically varying temperature can influence damage evolution. In this test, we assign a linear vertical temperature profile for each material point. The ice base temperature is set to −2°C, and the surface temperature is set to the value that yields the same depth‐averaged stiffness parameter, $\bar{B}$, from the isothermal case (approximately −16.7°C), where B is calculated from temperature according to the standard Arrhenius relation as given in Zwinger et al. (2007). To allow direct comparison with Figure 3c, we set $l_{c}$ = 1 km. The maximum principal damage field at calving corresponding to this temperature profile is given in Figure 5. Due to the warmer basal temperature, basal crevasses only propagate in the most stressed regions and the overall damage field is reduced outside of the rift. This reduced basal calving is likely more consistent with reality, where basal crevasses should only initiate from the center of the shelf under very high stresses. More commonly, flexural stresses, such as those experienced at the grounding line, are required to initiate basal crevasses (Logan et al., 2013), which we discuss further in Section 5.1. The ease with which temperature effects can be accounted for is an advantage of the GIMPM‐SSA creep damage model. Conversely, the zero‐stress model employed in the next two sets of experiments is formulated under the assumption of an isothermal ice shelf, and therefore, always overestimates the spatial extent of basal crevassing.

### 4.3 Zero‐Stress Damage Simulations

The zero‐stress criterion (Nye, 1957), states that in a closely spaced field of crevasses, each crevasse will propagate to the depth where the net longitudinal maximum principal Cauchy stress becomes zero. Although ice shelf crevasses are not typically closely spaced, the zero‐stress model was recast as an SSA isotropic damage variable by a previous study (Sun et al., 2017), where damage was defined as the ratio of the combined depths of surface and basal crevasses to the ice thickness (Sun et al., 2017). Here, we extend the zero‐stress damage variable to anisotropic form as a second‐order tensor, $\hat{D}$. We detail the anisotropic zero‐stress damage model and its implementation in Section S.2. To summarize, the zero‐stress model calculates 3‐D stresses using a similar effective pressure as Equations (15), (16), (17) used in the creep damage model, and ignoring the water pressure term for surface crevasses. However, the zero‐stress damage model is formulated in terms of applied stress and under the assumption that crevasses are in equilibrium with the stress field, where deviatoric stresses are considered depth‐invariant. Conversely, the creep damage model is updated in the rate form according to depth‐varying effective stress, which is sensitive to depth‐varying temperature and damage. Put simply, the zero‐stress model parameterizes crevasse depths only, while the creep damage function is a rate‐based parameterization of the progressive damage evolution process in 3‐D. A vertical damage profile for a column of ice according to the zero‐stress model resembles a step function, with maximum damage at depths where crevasses have propagated and zero damage elsewhere. A typical vertical profile using the creep damage model similarly shows maximum damage where crevasses have fully ruptured, but also exhibits non‐zero damage near the crevasse tips as well.

Here, we test the zero‐stress damage model on the MISMIP+ domain to demonstrate the impact of these differences in comparison to the creep damage results from Section 4.2. We run two experiments with the zero‐stress damage model, where each experiment tests the model in both fully isotropic and fully anisotropic form. To be consistent with the creep damage tests, we omit accumulation and melting entirely for both ice flow and its influence on zero‐stress damage until Section 4.4 when we test the modification proposed by Bassis and Ma (2015).

**Figure 6:**
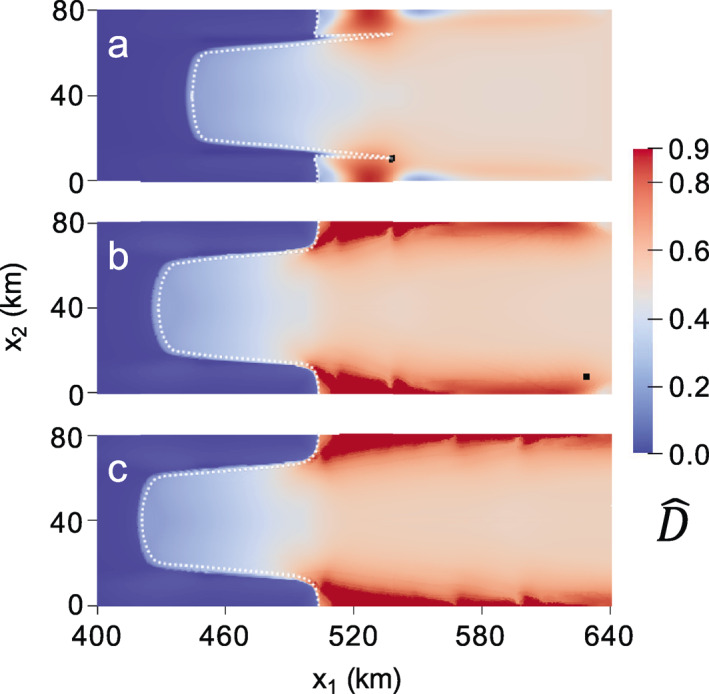
Isotropic zero‐stress damage field at (a) 0, (b) 16, and (c) 30 years. The black tracer particle highlights the highly advective flow regime. The white dotted line indicates the grounding line.

**Figure 7:**
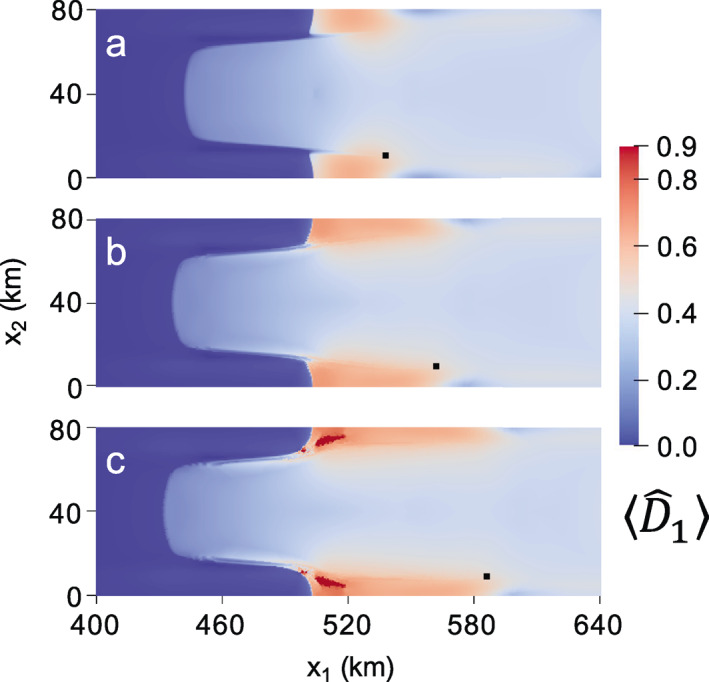
Fully anisotropic zero‐stress maximum principal damage field at (a) 0, (b) 16, and (c) 30 years. The black tracer particle highlights the highly advective flow regime.

In the first experiment, we run the zero‐stress damage model as given for 30 years. No critical rupture scheme is enforced. Note that this test was performed previously using the isotropic form over a longer time scale using the MISMIP+ geometry with the finite volume ice flow model Berkeley Ice Sheet Initiative for CLimate ExtremeS; Sun et al., 2017). The isotropic zero‐stress damage results near the grounding line are shown in Figure 6 at (a) 0, (b) 16, and (c) 30 years. At the first time step, damage immediately grows to $\hat{D}\;∼\;0.33$ near the grounding line and $\hat{D}\;∼\;0.5$ at the center of the ice shelf. With the exception of rifting, the zero‐stress and creep damage models generally agree concerning the spatial distribution of heavily versus weakly damaged areas. As was the case for creep damage, grounded ice experiences relatively little damage. Nearly ruptured ice immediately develops between the narrow strip of grounded ice at approximately $x_{1}\;∼$ 520 km and the lateral boundaries ($x_{2}=$ 0 and $x_{2}=$ 80 km). However, unlike in the creep damage case, this region does not develop into a sharp rift that propagates across the shelf to result in a calving event. Over time, the zero‐stress damage field mostly evolves from its initial configuration through advection, as evident following the black tracer particle in Figures 6a and 6b, which advects beyond the domain in Figure 6c. As expected, the damage field has a strong impact on the grounding line position (white dotted line) by decreasing buttressing to initiate grounding line retreat. This grounding line migration is reflected in the damage field, as ice that is nearing floatation quickly accumulates relatively heavy damage in comparison to upstream grounded ice. The corresponding anisotropic zero‐stress damage results are given in Figure 7, which yield lesser damage values everywhere compared to the isotropic case given that damage accumulation is restricted to a single plane. Like the isotropic case, anisotropic zero‐stress damage evolution is primarily dictated by advection, though relatively less advection occurs over the 30‐year simulation, as revealed by the black tracer particle in Figure 7 versus the isotropic case in Figure 6, because the lesser damage results in smaller velocities. While some material points eventually rupture by the end of the simulation, they do not result in tabular calving, even if the simulation is continued for several more decades. In agreement with Sun et al. (2017) none of the above zero‐stress simulations resulted in calving. We can conclude that the novelties of our approach, namely using a tensorial damage variable and implementing the model within the GIMPM‐SSA framework, are simply not enough to cause calving with the zero‐stress model in the MISMIP+ experiment.

**Figure 8:**
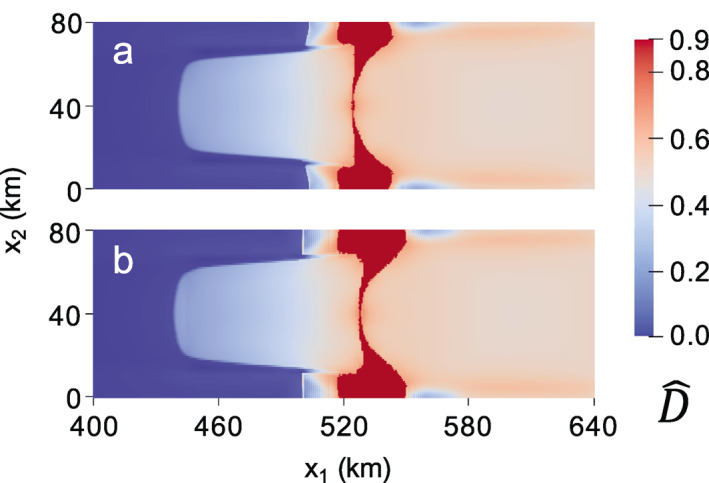
Isotropic zero‐stress damage field at calving when using ${\hat{D}}_{\text{cr}}$ = 0.7 for a grid resolution of (a) 0.5 versus (b) 1 km. Grid dependence is most apparent in the vastly different times to calving of (a) 0.553 versus (b) 1.607 years.

**Figure 9:**
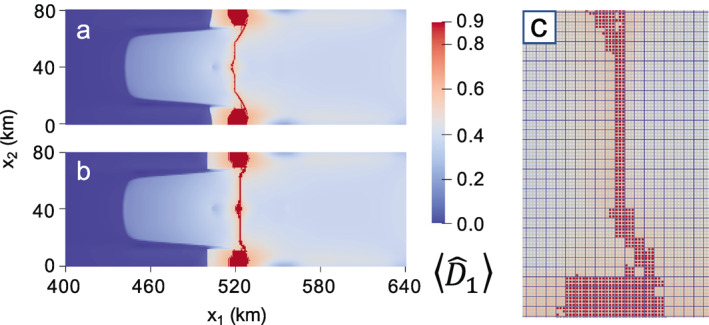
Fully anisotropic zero‐stress maximum principal damage field at calving when using ${\hat{D}}_{\text{cr}}$ = 0.6 for a grid resolution of (a) 0.5 versus (b) 1 km. The rifts propagate nearly instantly, with times to calving of (a) 5.73 and (b) 5.99 h. The rift paths show clear grid dependence, as shown in detail (c) for the 1‐km case.

In the second zero‐stress damage experiment, we rerun the MISMIP+ experiment, but encourage rifting to initiate by setting critical damage values of ${\hat{D}}_{\text{cr}}=\;0.7$ and ${\hat{D}}_{\text{cr}}=\;0.6$ for isotropic and anisotropic damage, respectively. While the choice of these values is rather *ad hoc*, they allow for the formation of sharp rifts. The critical rupture criterion is enforced after each combined zero‐stress damage and SSA solution. At the first time step, rupture occurs near the shear margins where $\langle {\hat{D}}_{1}\rangle >{\hat{D}}_{\text{cr}}$, and the resulting high stresses allow rifts to propagate across the domain to calve tabular icebergs. The final maximum principal zero‐stress damage fields are given in Figures 8 and 9 for the isotropic and anisotropic cases, respectively. While both cases produce rifts in the same general area as the creep damage experiments, this test exposes how, as a local damage model, the zero‐stress damage model is subject to grid dependence. Figures 8a and 9a use a 0.5‐km grid resolution whereas Figures 8b and 9b use a 1‐km grid resolution. Strong grid dependence is observed in the spatial damage field for the anisotropic case. The differing grid resolution results in different rift paths, where rifting clearly localizes to single grid cells, as shown in detail for the 1 km resolution case in Figure 9c. In the isotropic case, grid resolution also influences the rift width. However, the strongest grid dependence in the isotropic case is associated with the time to calving; the 0.5‐km resolution grid results in calving in 0.553 versus 1.607 years for the 1 km resolution grid.

We conclude that there are three main disadvantages to the zero‐stress damage model which are not present in the nonlocal creep damage model: (a) It is formulated on the basis of estimating depths of closely spaced crevasses, which is not appropriate for modeling rifting or ice shelf crevasses, which tend to be widely spaced; (b) the zero‐stress damage evolution exhibits grid dependence, which especially affects both the path and rate of rift propagation; and (c) deviatoric stresses are assumed to be constant with depth, so that variations in temperature and damage with depth are not accounted for, which typically results in an overestimation of basal crevassing. We further caution that there may be additional numerical challenges when working with the zero‐stress damage model concerning stability and time‐stepping. To ensure stability in the zero‐stress damage response, we implement a similar adaptive time‐stepping scheme as in the creep damage model, where time steps are adjusted with the goal of achieving a maximum change in 2‐D damage between time steps of $\delta_{2}=0.05$ (Section S.2). This scheme resulted in time steps as small as fractions of a second in the anisotropic zero‐stress rifting simulation, where damage accumulation was so rapid that it took only 6 h to calve a tabular iceberg. Such small time steps, therefore, restrict timescales that are computationally feasible to model. Without a reference solution, we cannot definitively say whether this modeled rapid rift propagation rate is incorrect. However, observed rift propagation is typically much slower, where it takes months or years for rift propagation to cause calving.

### 4.4 Simulations Using the Modification for Necking and Mass Balance

A drawback of both the creep and zero‐stress damage models as tested above is that they do not account for the potential impact that processes associated with necking and mass balance may have on damage evolution. In Section S.3, we explain how these processes influence crevasse depths, and we describe an expression (Equation S2) that modifies large‐scale damage to account for these processes (Bassis & Ma, 2015). In this section, we implement this expression only within the zero‐stress damage model, noting that its implementation within the creep damage model is less straightforward (see Section 5.1), and is beyond the scope of this study. By comparing the results from this modified zero‐stress damage model to those from the standard zero‐stress damage model, we can analyze how necking and mass balance processes impact damage. Thus, we can determine the settings in which our creep damage model is applicable in its current form without accounting for these processes, and then propose how a combined approach between damage models may be formulated for more generalized applications.

**Figure 10:**
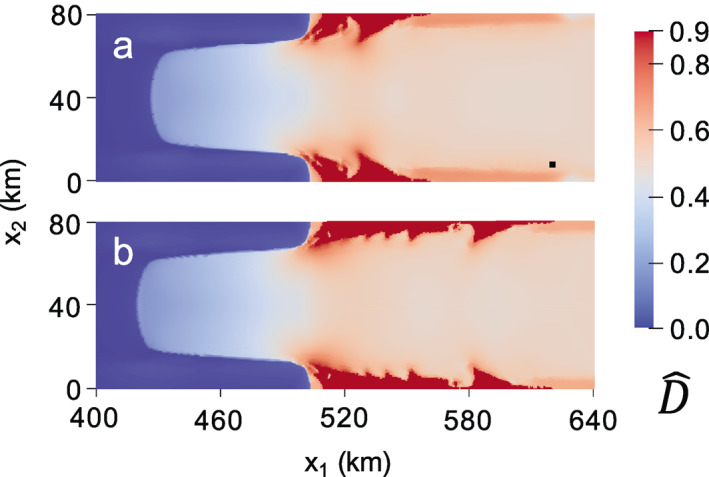
Isotropic zero‐stress damage field, as modified to include necking, at (a) 16, and (b) 30 years. The initial field at 0 years is identical to Figure 6a.

**Figure 11:**
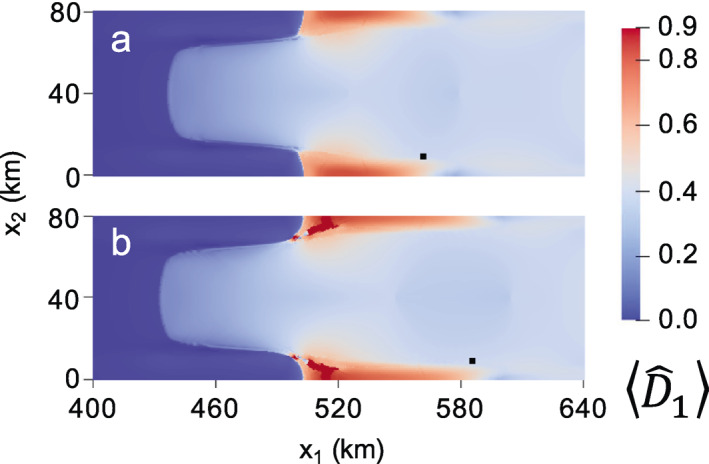
Fully anisotropic zero‐stress maximum principal damage field, as modified to include necking, at (a) 16, and (b) 30 years. The initial field at 0 years is identical to Figure 7a.

We perform two experiments with the modified zero‐stress model. Both experiments resemble the first experiment from the previous section, where the damage model is activated and the MISMIP+ model is run forward in isotropic and anisotropic form for 30 years. For the first experiment, we set melting and accumulation to zero, so that when the modified and standard zero‐stress damage results are compared, the role of necking alone is revealed. The results for the necking‐only experiment are shown in Figures 10 and 11 for the isotropic and anisotropic cases, respectively. In these figures, the first time step is not shown because it is the same as in the standard cases (Figures 6a and 7a, respectively). Like the standard case, the modified zero‐stress model yields large values of damage around the farthest downstream grounding line portions of the ice shelf margins, and this damage advects toward the ice front over time. Near these heavily damaged areas, the ratio of gravitational restoring stress to tensile stress, represented by $S_{0}$ in the necking model (see Section S.3), is less than one, so that necking accelerates the rate of damage accumulation. Elsewhere, the gravitational restoring stress exceeds tensile stress ($S_{0}>1$), so that damage healing occurs. Due to this trend of accelerated damage in some areas, with healing in the immediately surrounding areas, rifting in the modified isotropic case develops into sharper patterns than in the standard isotropic case. However, rifts still do not propagate across the center of the shelf. Sharper damage patterns also occur in the modified anisotropic case as compared to the standard anisotropic case, especially near the lateral boundaries, though rifting is similarly minimal in both cases. Interestingly, the configuration of fully damaged material points in the isotropic modified zero stress simulation (Figure 10b) resembles the sawtooth pattern of calving sometimes observed at the lateral sides of long ice tongues (e.g., Erebus ice tongue).

Generally, the necking expression only yields additional damage accumulation along areas where stress is already elevated, with healing dominating the response elsewhere. However, upon healing, many regions of the domain quickly re‐damage toward their previous values. For example, the ice tongue part of the domain is mostly under uniaxial tension, which in the isotropic case, yields the expected values of $\hat{D}\approx 0.5$ and $S_{0}\approx 2$. Any healing from the necking model is immediately countered by new zero‐stress damage accumulation during the next computational cycle. However, at the locations where the ice tongue inherits heavy damage from upstream along the lateral bounds in the standard, isotropic case (Figures 6b and 6c), healing is observed in the modified case that is maintained over time (Figure 10). In the anisotropic case (Figure 11), sustained healing is more apparent along the upstream shear margins of the ice shelf.

**Figure 12:**
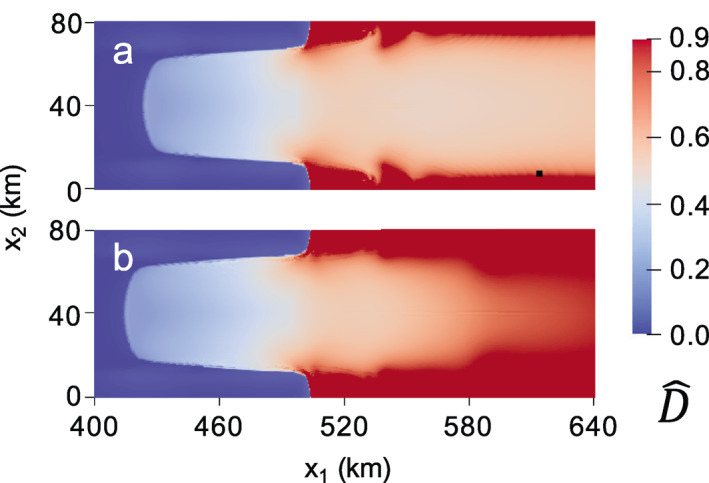
Isotropic zero‐stress damage field, as modified to include necking and 5 m a^−1^ basal melting for floating ice, at (a) 16, and (b) 30 years. The initial field at 0 years is identical to Figure 6a.

**Figure 13:**
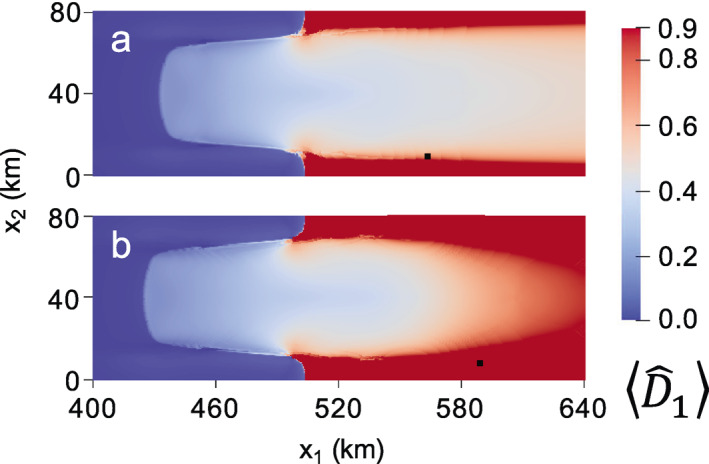
Fully anisotropic zero‐stress maximum principal damage field, as modified to include necking and 5 m a^−1^ basal melting for floating ice, at (a) 16, and (b) 30 years. The initial field at 0 years is identical to Figure 7a.

For the second modified zero‐stress experiment, we test the impact of assigning a basal melt rate. We rerun the first experiment with a basal melting rate of 5 m a^−1^, which is taken as constant throughout the floating ice domain, for simplicity. The isotropic and anisotropic results are given in Figures 12 and 13, respectively, and we note that setting a greater or lesser basal melting rate yields similar patterns. For the isotropic case, the damage field toward the interior of the floating domain at 16 years (Figure 12a) is very similar to the necking‐only case (Figure 10b), because the increase in damage due to basal melting is generally unable to offset the decrease in damage due to healing in this area. The opposite effect occurs near the thin, lateral bounds of the floating domain, and maximum damage is quickly realized. By the end of the simulation (Figure 12b), enough thinning has occurred throughout the floating domain that melt‐induced damage begins to dominate over healing for more interior sections of the ice tongue. A similar response is observed in the anisotropic case (Figure 13), except that at the interior sections of the ice tongue, melt‐induced damage overtakes healing earlier in the simulation than in the isotropic case. Healing in this area is lesser for the anisotropic case than the isotropic case. We can conclude that for both the isotropic and anisotropic modified zero‐stress cases, the addition of widespread basal melting increases damage primarily along the thin, lateral boundaries, but it does not result in any sharp rifting that results in tabular calving.

## 5 Discussion

### 5.1 Impact of Necking and Mass Balance Effects on Damage Evolution

The experiments from Section 4.4 indicate that necking and mass balance may play significant roles in modulating damage on decadal timescales, so that these processes should be implemented within the creep damage model if it is to be applied on long timescales (decades to centuries). Such an approach will be the subject of future research, and would require modifying the 3‐D damage field to reflect the modified value of vertically integrated damage calculated according to necking and mass balance. This process could include adjusting the vertical coordinates and local damage values of each layer, as well as the addition or subtraction of layers. Based on our previous comparison between creep damage and zero‐stress damage, we would expect a combined creep‐damage/necking model to behave somewhat differently than the combined zero‐stress damage/necking model. While incorporating necking effects simply sharpened the zero‐stress damage field in regions of elevated stress, this sharpened damage could develop into rifting with the creep damage model that would otherwise not occur. Similarly, targeted basal melting could also trigger additional rifting. However, we emphasize that necking and mass balance effects should not always be necessary to initiate rifts, according to LEFM rift studies (e.g., Lipovsky, 2020). Encouragingly, the creep damage model can initiate realistic rifting without these additional effects (Section 4.2), though we acknowledge that given the idealized setting, it is difficult to determine whether or not this rifting should actually occur.

The major advantage of combining the Bassis and Ma (2015) model with creep damage concerns healing. Basal crevasses are typically initiated near the grounding line or perturbations such as ice rises, and can heal heavily as they advect downstream, due to both gravitational restoring stresses and marine ice formation. Healing of upstream damage has been inferred, for example, on Larsen C Ice Shelf (Borstad et al., 2013). Healing in the modified zero‐stress experiments was probably underestimated; most healing was immediately offset by new damage because the zero‐stress model assumes crevasse depths are in equilibrium with the stress field. This effect was likely amplified by the assumption in the zero‐stress damage model that deviatoric stresses are depth‐invariant, which likely causes basal crevassing to be overestimated. However, creep damage is rate‐based and can incorporate 3‐D temperature and stresses. As seen in Figure 5, when lower basal temperatures are accounted for, basal crevasses do not spontaneously propagate in low stress regions at the interior of the ice shelf. Therefore, when using a combined creep‐damage/necking model with mass balance effects, damage associated with deep basal crevasses that were initiated from high stress regions upstream could become completely healed in low stress regions downstream. However, the success of capturing this behavior is reliant on proper initiation of the damage field corresponding to upstream basal crevasses.

In the case that basal crevasses initiate from flexural stresses at the grounding line, special treatment is required to initiate the corresponding damage because such stresses are not captured in the SSA. The simplest approach may be to assign a 3‐D damage distribution according to crevasse depths calculated with the SSA zero‐stress approximation. However, this approach would be strictly a rough approximation, as for example, the zero‐stress model was found to significantly underestimate basal crevasse depths near the grounding line on Larsen C Ice Shelf where flexural stresses are large (Luckman et al., 2012). These authors found better agreement with observations (within 10%–20%) when using a LEFM approach, though this approach also did not explicitly account for flexural stresses and may not be accurate in all cases. An approach for approximating basal crevasse depth at the grounding line that does account for flexure involves using a thin elastic beam approximation, combined with a mode I brittle failure criterion (Logan et al., 2013), but this model is only applicable where strain rates are low. In addition, flexure associated with ungrounding of highly lubricated ice streams can likely be accounted for by combining the SSA with a thin‐plate treatment of viscoelastic flexure (MacAyeal et al., 2021); this model can also account for flexure away from the ice shelf boundaries (e.g., flexure associated with ice rumples, supraglacial lakes, and sea swells). The most accurate way of capturing flexural stresses associated with ungrounding may be to transition to a full‐Stokes model near the grounding line, though this approach is extremely computationally expensive in 3‐D. LEFM could be used to obtain reasonable basal crevasse heights in a full‐Stokes 2‐D flowline model (Yu et al., 2017), or the creep damage model could potentially be applied in a full‐Stokes setting as well.

### 5.2 Potential Modifications to the SSA Creep Damage Parameterization

For simplicity, we implemented the Keller and Hutter (2014) SSA creep damage parameterization largely as given, with only minimal modification to accommodate our use of a tensorial damage variable. However, we acknowledge that there are several potential sources of error in how the approximated 3‐D stress field is calculated, using the effective pressure from Equations (15), (16), (17), which should be considered in future development of the model. First, approximating effective pressure by superposing basal water pressure with ice pressure is only truly physically valid under LEFM assumptions, that is, linear elastic rheology and sharp cracks, but we assume viscous rheology and diffuse cracks. Furthermore, in Equation 17 it is assumed that basal water pressure is zero for ice grounded above sea level, which may not be true in all cases. Despite these deficiencies, the Keller and Hutter (2014) parameterization appears to be qualitatively accurate enough to allow realistic damage patterns to develop, and uses a similar water pressure parameterization as the SSA zero‐stress damage model and LEFM models (e.g., Nick et al., 2010; Sun et al., 2017; van der Veen, 1998b), facilitating comparison between models in this study. We note that within a full‐Stokes setting, water pressure has been incorporated into damaged ice using a poromechanics approach (Duddu et al., 2020; Mobasher, et al., 2016). A similar approach could potentially be adapted for the SSA parameterization, which we plan to consider in our future work.

The approximated 3‐D stress also neglects the influence of processes such as necking, buoyancy forces, crevasse shielding, and rounding off of crack tips due to melt or accretion of new ice. Additionally, it neglects flexure that occurs everywhere along rift walls (Lipovsky, 2020). Although observational evidence suggests that rift propagation is primarily controlled by horizontal glaciological stresses from creep flow (Joughin & MacAyeal, 2005), flexure along rift walls may play a role in rift propagation, for example, by inducing contact between rift flanks (Lipovsky, 2020). This process should be investigated further in future work. Our model also neglects similar flexure at the ice front that results in calving of small icebergs with areas on the order of hundreds of m^2^ to a few km^2^ (e.g., Christmann et al., 2019). However, icebergs with horizontal areas over 5 km^2^ account for about 95% of the calved ice mass in Antarctica (Tournadre et al., 2016). This suggests that small‐scale calving is relatively unimportant compared to the rift‐driven, large, tabular calving events that our model can simulate. Lastly, we acknowledge that by using an isotropic flow law, we do not account for anisotropic ice fabric, which may influence damage accumulation and rift propagation. It may be possible to parameterize an anisotropic flow model (e.g., Ma et al., 2010) for the SSA, where anisotropic fabric evolution and viscosity would be calculated in 3‐D similarly to the SSA damage parameterization.

Other improvements to the SSA creep damage parameterization may be possible concerning the conversion between applied and effective stress. Recall that Keller and Hutter (2014) only scale the deviatoric applied stresses by damage to calculate the effective stress, under the assumption that volumetric stress should not be affected by damage because it is mostly compressive in the SSA limit. These authors acknowledge that this assumption may underestimate a small amount of damage evolution near the surface, where tensile volumetric stress may arise. Therefore, it may be appropriate to forgo this assumption wherever volumetric stress is tensile, and instead calculate the effective stress by scaling the entire applied stress tensor by damage according to Equation 6, and evaluate the Hayhurst criterion according to Equation 10. In fact, it may be appropriate to consider this alternative effective stress parameterization at all depths as long as $\langle D_{3}\rangle$ is always set to zero in accordance with the compressive overburden stress. Implementing this alternative approach at all depths yields very similar damage patterns as the Keller and Hutter (2014) approach, as shown in Figure S3 for the fully anisotropic case.

One of the most significant advancements made with the creep damage framework presented here is in modeling and simulating the initiation and propagation of rifts using damage. While it is encouraging that our simple isotropic rift treatment clearly propagates rifts, our ongoing research efforts are aimed at enabling a more accurate physical depiction of rift dynamics. Ideally, rifts that open into the ocean should be considered as an evolving ice‐ocean interface, with the Neumann boundary condition assigned along the flanks similar to the ice front boundary condition, but which also includes any opposing pressure of ice mélange within the rift and partial contact between rift flanks (Larour et al., 2014; Lipovsky, 2020). Using material point methods, this boundary condition could potentially be applied directly on material points in a similar manner to how water pressure has been incorporated into full‐Stokes creep damage simulations (Duddu et al., 2020). Alternatively, it could be applied along line segments that are introduced to track cracks, and which can advect with flow (Nairn, 2003). Once rift boundary treatment is implemented, behavior of ruptured material points can be further modified to account for the strength of mélange between flanks, tension/compression asymmetry, and lateral friction or faulting between flanks.

Finally, compressibility of ice is also important to consider in future work. Damage accumulation is associated with a small volumetric increase (Sinha, 1991); however, Pralong and Funk (2005) neglected this increase and assumed that damaged ice remains incompressible. This assumption allows use of the strain equivalence principle (Section 2.2), which implicitly postulates that the damaged ice remains incompressible. However, fully damaged regions should be ideally treated as compressible. In the full‐Stokes formulations of Duddu et al. (2020) and Jiménez et al. (2017) the incompressibility constraint was not enforced in fully damaged regions, which improved the evaluation of stress near the crack tips. Furthermore, the evolution of damage and eventual opening of cracks (i.e., rifts and crevasses) changes the local body force field and the stress field due to density changes. In future extensions of the proposed SSA damage formulation, we intend to relax the incompressibility constraint and incorporate density changes in damaged regions.

### 5.3 Tuning of the Damage Model With Experimental and Observational Data

It is possible that the creep damage model parameters, including $B^{*}$, *r*, *α*, *β*, and γ, are dependent on physical variables, such as temperature, grain size and orientation (fabric), and interstitial liquid water content. Pralong and Funk (2005) note that the Hayhurst weights can be approximately constant for temperatures near the melting point (where the change of brittle‐ductile ice behavior is small), which may be appropriate for ice shelves. Duddu and Waisman (2012) calibrated a simplified temperature‐dependent form of the creep damage model for ice, but the calibration used a single set of uniaxial compression data from Jacka (1984). Owing to the scarcity of experiments, observations, and prior modeling studies, there are no functional forms describing the dependencies of the damage parameters on the various physical variables. While we assumed these parameters as constants, this limitation can be overcome if new experimental or observational data becomes available.

In Chapter 4 of Huth (2020), we used the model along with observational data on Larsen C ice shelf (and the calving of iceberg A68 in 2017) to calibrate the damage parameters and assess which parameters influence rift propagation. We find that the damage parameter $B^{*}\;$ can be simply tuned to match the rift propagation rate, whereas the parameter ${\bar{D}}_{\max}$ can be tuned to match the propagation path. We also find that the modeled Larsen C rift propagation path is less sensitive to the weights associated with brittle‐ductile failure modes (*α* and *β*) in the Hayhurst criterion, assuming the weight associated with effective pressure (λ) is small. Aside from this, we recently developed a two‐parameter phase field damage model (Sun et al., 2021) based on LEFM that can replace the creep damage model and reduce parametric uncertainty. We use the creep damage model as it is simple and performs reasonably well, but the proposed MPM‐SSA modeling framework can easily ingest other more appropriate damage models. We acknowledge that for our modeling framework to be predictive and not simply descriptive it is important to minimize parametric sensitivity and establish a robust methodology for parameter calibration, which will be the focus of future work.

## 6 Conclusion

In this study, the mechanical weakening and fracture of large‐scale ice shelves is modeled using an SSA parameterization for nonlocal, anisotropic creep damage. The creep damage model parameterizes the fracture process as time‐dependent accumulation of damage; consequently, it allows for the development of well‐defined rift‐like features resembling those observed on the floating portion of the Pine Island Glacier (Alley et al., 2019; Jeong et al., 2016). In contrast, the zero stress model parameterizes the fracture process based on crevasse depth estimates and does not produce a well‐defined rift in the MISMIP+ example. This illustrates that the creep damage approach can provide a better parameterization for simulating rift propagation in real ice shelves. Furthermore, creep damage is treated in 3‐D, which allows damage interaction with other 3‐D variables, such as temperature and density. The numerical framework that we built to support the creep damage model is formulated using the GIMPM, which allows accurate and efficient advection of the 3‐D damage field. In contrast, if the model was implemented within a traditional Eulerian framework, advection algorithms would be computationally inefficient, and introduce numerical diffusion error that would compromise the accuracy of damage evolution over long timescales.

By testing the creep damage model on an idealized marine ice sheet, we find that large‐scale damage of ice should be treated as fully anisotropic. Anisotropic creep damage yields sharper, more arcuate rifting and crevasse patterns that are more consistent with observations. In addition, anisotropic nonlocal damage is thermodynamically consistent (Pralong et al., 2006) and mesh size independent (Duddu & Waisman, 2013), even though it is not based on thermodynamic arguments relating fracture energy and strain energy release rate, as in the phase field damage model (Sun et al., 2021). Our experiments further show that deep crevassing, rifting, and tabular calving may occur using creep damage without the inclusion of necking or mass‐balance processes. Testing a modified form of the zero‐stress damage model that includes these processes (Bassis & Ma, 2015) does not capture rifting that results in calving, unless an *ad hoc* critical damage is defined. Therefore, we conclude that the failure of zero‐stress damage approaches to capture rifting does not occur due to the absence of these processes, but because the zero‐stress model inappropriately assumes that crevasses are in equilibrium with the stress field; moreover, it and suffers from numerical issues related to its local damage formulation. While our ongoing research is focused on the calibration/validation of the damage parameters with an application to real ice shelves (e.g., Larsen C), and improved representation of rift boundary conditions, in our future work we intend to combine the necking/mass‐balance and creep damage models for an ideal representation of ice‐shelf fracture on decadal timescales.

## Supporting information

Supporting Information S1

## Data Availability

The simulations in this study can be reproduced using the experimental setups and model source code available at https://doi.org/10.5281/zenodo.4657848.
