# Supplementary material for: A Generalized Interpolation Material Point Method for Shallow Ice Shelves. 2: Anisotropic Nonlocal Damage Mechanics and Rift Propagation
Source: J Adv Model Earth Syst. 2021 Aug 24;13(8):e2020MS002292. doi: 10.1029/2020MS002292 (PMC8459271; doi:10.1029/2020MS002292)
Supplement: Supplementary file 1 — Supporting Information S1 [file JAME-13-e2020MS002292-s001.pdf]

AGU PUBLICATIONS

Journal of Advances in Modeling Earth Systems

Supporting Information for

A generalized interpolation material point method for shallow ice shelves. Part II: anisotropic nonlocal damage mechanics and rift propagation

A. Huth[1], R. Duddu[2,3], and B.E. Smith[4]

[1]Department of Earth and Space Sciences, University of Washington, Seattle, WA, USA, [2]Department of Civil and Environmental Engineering, Vanderbilt University, Nashville, TN, USA, [3]Department of Earth and Environmental Sciences, Vanderbilt University, Nashville, TN, USA, [4]University of Washington, Applied Physics Laboratory, Polar Science Center, Seattle, WA, USA

Contents of this file

S.1 Table S1 and Supporting Figures S1 – S3
S.2: Description of zero-stress damage model
S.3: Description of damage modification for necking and mass balance

Introduction

In Section S.1 of this supporting information, the early MISMIP+ creep damage accumulation for isotropic ($\gamma = 0$) and mixed isotropic/anisotropic creep damage ($\gamma = 0.5$) are reported at similar levels of rift propagation as given for fully anisotropic creep damage ($\gamma = 1$) in Figure 2 of the main text. In addition, the damage field at calving is given for the fully anisotropic creep damage case where the entire Cauchy stress tensor is scaled by damage, rather than only scaling the deviatoric components. Further description and implementation details of the SSA zero-stress damage model (Sun et al., 2017) and the necking and mass balance modification (Bassis & Ma, 2015) are given in Sections S.2 and S.3, respectively

S.1 Supplementary Tables and Figures

Table S1: MISMIP+ parameters (Asay-Davis et al., 2016)

Parameter	Value	Description
$\dot{a}$	0.3 m a^{-1}	mass balance rate (spinup only)[†]
n	3	Glen's law exponent
ρ_w	1028 kg m^{-3}	Density of seawater
ρ	918 kg m^{-3}	Density of ice
g	9.81 m s^{-2}	Acceleration of gravity
z_{sl}	0 m	Sea-level elevation
$\bar{B}$	1.16×10^8 Pa $s^{1/3}$	Glen's law vertically-averaged stiffness parameter
$(\alpha^*)^2$	0.5	Coulomb law friction coefficient
m^*	3	Friction-law exponent

[†]For all damage experiments, $\dot{a}$ is set to 0 m a^{-1}, except when basal melt is applied in Section 4.4, where $\dot{a} = -5$ m a^{-1}.

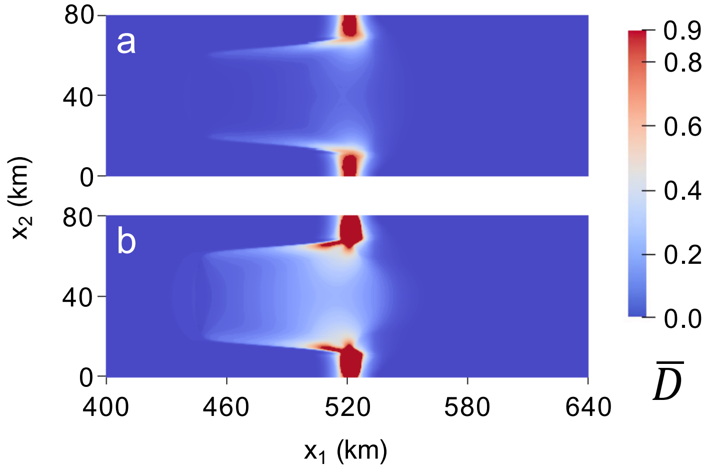

Figure S1. Damage field for the isotropic ($\gamma = 0$) creep damage simulation at (a) 0.06 years and (b) 0.12 years. Material points with $\bar{D} = \bar{D}_{\max} = 0.9$ correspond to rifts.

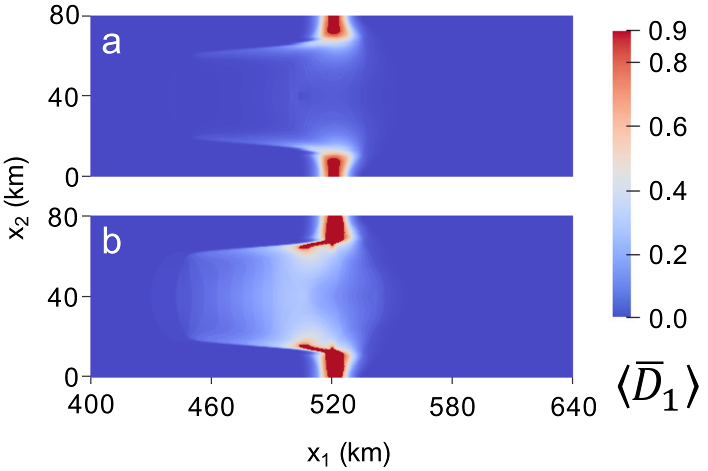

Figure S2. Maximum principal damage field for the mixed isotropic/anisotropic ($\gamma = 0.5$) creep damage simulation at (a) 0.06 years and (b) 0.18 years. Material points with $\langle \overline{D}_1 \rangle = \overline{D}_{\text{max}} = 0.9$ correspond to rifts.

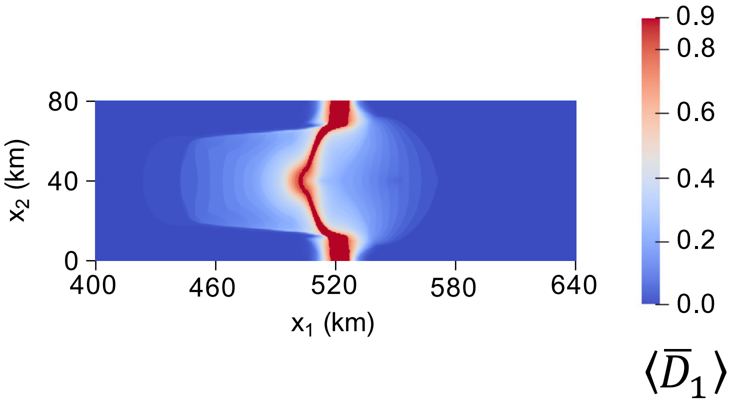

Figure S3. Maximum principal damage field at 0.51 years for the fully anisotropic ($\gamma = 1$) creep damage simulation where effective stress is calculated by scaling the entire applied Cauchy stress tensor by damage (Equation 6), rather than only scaling the deviatoric stress by damage (Equation 18) as in Keller and Hutter (2014).

S.2 Description of zero-stress damage model

In the zero-stress criterion, closely-spaced crevasses are assumed to propagate to the depth at which the net longitudinal maximum principal Cauchy stress is zero (Nye, 1957). The net Cauchy stress at depth is parameterized as

$$\sigma_{ij}(z) = \sigma_{ij}^{\mathrm{D}}(z) - p_{\mathrm{eff}}(z)\delta_{ij}\,, \tag{S1}$$

where $p_{\mathrm{eff}}(z)$ takes the same form as within the creep damage model from Equations (15)-(17). We disregard the water pressure term for surface crevasses and assume dry surface conditions. A zero-stress isotropic damage variable was previously defined for SSA models as the ratio of the combined depths of surface and basal crevasses to the ice thickness (Sun et al., 2017), and here, we extend this damage variable to anisotropic form as a 2nd order tensor, $\widehat{\boldsymbol{D}}$. To our knowledge, all other SSA applications of the zero-stress model have solely focused on obtaining plausible estimates of crevasse depths (Pollard et al., 2015; Bassis & Walker, 2012; Bassis & Ma, 2015), rather than also applying the crevasse depths as a damage variable that influences the stress solution. Zero-stress crevasse depths are assumed to be in equilibrium with the stress field, and given the interdependence between damage and stress, the zero-stress damage solution must therefore be computed simultaneously with the SSA solution. This coupled solution is facilitated by assuming deviatoric stresses are depth-invariant, which allows an analytical solution for crevasse depths (Nick et al., 2010). We adopt this assumption for simplicity, as did the previous SSA zero-stress damage study (Sun et al., 2017). However, assuming depth-invariant deviatoric stresses is only justified only if crevasses are closely-spaced so that the stress singularity at crevasse tips is dissipated (Weertman, 1977), and if vertical ice columns are isothermal.

We emphasize that the zero-stress approximation is likely more accurate when applied to outlet glaciers in Greenland (e.g. Nick et al., 2010; Todd & Christofferson, 2014) than when applied to ice shelves, where the assumptions of closely-spaced crevasses in equilibrium with the stress field and crevasse evolution based on only tensile stresses are less valid. Ice shelf basal crevasses tend to be widely-spaced and may experience mixed-mode fracture (McGrath et al., 2012; Luckman et al., 2012). Furthermore, assuming an isothermal ice shelf may not be an accurate approximation, as seawater temperatures at the ice shelf base greatly exceed surface air temperatures. However, a vertically-varying temperature profile would induce vertically-varying deviatoric stresses, so that a more complex iterative scheme would be required here solve the coupled SSA/zero-stress damage problem.

We restrict our zero-stress damage tests to the fully-isotropic and fully-anisotropic cases. For full-anisotropy, the initial damage accumulation for the zero stress model occurs on a single plane aligned normal to the maximum principal stress of the undamaged configuration, as in the creep damage model. This plane subsequently rotates over time according to spin, as in Equation (8). However, unlike creep damage, anisotropic zero-stress damage accumulation must be restricted to this plane at subsequent time steps, and evolves according to the stresses normal to the plane because the zero-stress criterion assumes crevasses open in accordance with tensile (Mode I) fracture. Rifting is incorporated with the same 2-D critical damage rupture scheme from the creep damage model. For stability, we attempt to restrict the change in zero-stress damage between time steps using the same adaptive time-stepping scheme used for the creep damage simulations, but defining $\overline{\mathrm{dD}}_{\mathrm{max}} = \max\left(\widehat{D}^{m+1} - \widehat{D}^{m}\right)$. Note that for zero-stress damage, we must eliminate the condition to restart the damage solution if $\overline{\mathrm{dD}}_{\mathrm{max}} > 0.075$ because damage is solved implicitly; however, we still achieve stable simulations with sharp

rifting because the next time step is always adjusted with the goal of achieving $\overline{\mathrm{dD}}_{\max} = \delta_2$, where we set $\delta_2 = 0.05$.

S.3 Description of damage modification for necking and mass balance

Necking describes the process in which basal crevasses widen under tension and the resulting feedback on crevasse evolution, where depending on strain-rates and crevasse-geometry, the ratio of crevasse penetration to ice thickness (i.e. damage) will either increase or decrease over time (Bassis & Ma, 2015). The ratio can increase due to greater thinning rates associated with the presence of crevasses. However, as crevasses grow, the local ice geometry simultaneously adjusts to hydrostatic equilibrium, and depressions fill with surrounding ice due to "gravitational restoring" stresses. If the system is dominated by these gravitational stresses rather than thinning, the ratio of crevasse penetration to ice thickness will decrease (i.e. healing). The ratio is further modulated by mass balance processes, such as melting and accumulation of snow or marine ice in crevasses. A previous study investigated this complex coupling of various processes, and an expression for large-scale ice flow was proposed using perturbation analysis that defines the rate at which damage is modulated according to necking and mass balance processes (Bassis & Ma, 2015). This model can be employed in conjunction with a mechanical damage model that tracks crevasse depths, but has not yet been tested to our knowledge.

When used in conjunction with the zero-stress model, this large-scale damage modification takes the form:

$$\frac{d\widehat{\boldsymbol{D}}}{dt} = \left(n^*(1 - S_0)\langle\dot{\varepsilon}_1\rangle + \frac{\dot{m}}{H}\right)\widehat{\boldsymbol{D}}\,, \qquad \text{(S2)}$$

where the first term in the parentheses describes the influence of necking on damage and the second term describes the influence of the melt rate, $\dot{m}$. Within the necking term, parameter n^* is an effective flow law exponent and S_0 describes the ratio of gravitational restoring stress to tensile stress. Derivation of these terms is non-trivial, and we direct the reader to the original publication for a detailed explanation. The expression is only valid in the long wavelength limit, which corresponds to the following assumptions: crevasses are wide compared to the ice thickness, perturbations are assumed to relax immediately to hydrostatic equilibrium, and the melt rate in crevasses is equivalent to the large scale melt rate. We solve (S2) immediately after completion of the SSA solution, and add the damage increment to the zero-stress damage calculated during the SSA.
